# The potential of red‐fleshed apples for cider production

**DOI:** 10.1111/1541-4337.70167

**Published:** 2025-04-04

**Authors:** Marbi Schwartz, Dalene de Beer, Jeannine Marais

**Affiliations:** ^1^ Department of Food Science Stellenbosch University Stellenbosch South Africa; ^2^ Sensory Department HEINEKEN Beverages Stellenbosch South Africa; ^3^ Plant Bioactives Group, Post‐Harvest and Agro‐Processing Technologies Agricultural Research Council (Infruitec‐Nietvoorbij) Stellenbosch South Africa

**Keywords:** anthocyanins, major volatile compounds, phenolic compounds, physicochemical, sensory

## Abstract

Cider quality is influenced by numerous factors relating to the apples used during production. While extensive research has been done to explore the phenolic content, sensory quality, and storage stability of various apple products, the domain of fermented apple products, such as ciders, remains underrepresented. Red‐fleshed apples (RFAs) have naturally high concentrations of phenolic compounds, which indicate their potential in the production of novel cider products. However, a knowledge gap remains regarding the application of RFAs in cider production and how their physicochemical and sensory properties are changed during processing. This review is the first to comprehensively investigate whether and to what extent apple categories (dessert, cider, and RFAs) differ regarding their physicochemical and sensory properties from harvest throughout cider processing. Furthermore, it highlights the importance of a holistic understanding of apple characteristics, encompassing both traditional and RFA varieties in the context of cider production. The findings offer valuable insights for stakeholders aiming to enhance product quality, providing a foundation for future studies on optimizing processing methods for a diverse and appealing range of ciders.

## 1 INTRODUCTION

Apples are one of the most widely cultivated fruits in all temperate regions of the world, playing an important role in the global economy (Alberti et al., 2016; Bhat et al., 2023). Apples are mainly produced for the fresh market, but a substantial amount is also processed into food products (i.e., dried products and jams) and beverages (i.e., juices, concentrates, and ciders) (Feng et al., 2021; Guyot et al., 2009; Nicolas et al., 1994; Soomro et al., 2022). Modern‐day plant breeding programs aim to create apple varieties that satisfy the criteria of various supply chain participants and consumers (Sestras & Sestras, 2023). Furthermore, the biodiversity and chemical diversity, related to the sensory properties, of apples hold substantial potential for customizing processed products to specific consumer preferences (Chitarrini et al., 2021).

The investigation and use of colored fruits and vegetables have become more prevalent, further expanding and diversifying the fresh and processed fruit markets (Bars‐Cortina et al., 2017). Additionally, the expansion of the cider industry increased the demand for apple varieties with relatively high concentrations (>1000 mg/kg fresh weight [FW]) of phenolic compounds (Jalali et al., 2024; Thompson‐Witrick et al., 2014). Red‐fleshed apples (RFAs) are nontraditional apple varieties valued for their red juice with high phenolic concentration, especially anthocyanins (Espley et al., 2013). The phenolic content is of particular interest due to its contribution to cider color, bitterness, and astringency (Alonso‐Salces et al., 2004; Laaksonen et al., 2017). Although RFAs are historically bred for fresh consumption, they show exciting potential for cider making.

Cider production is governed by various safety and quality regulations to ensure the final product is safe for consumption and maintains high‐quality standards (Rosend et al., 2020). These regulations, which differ by region, are designed to prevent microbial contamination, ensure consistent product quality, and comply with regional food safety laws. Similarly, any new apple variety aiming to compete with or replace varieties currently available in the market (either for fresh consumption, juicing, or cider production) must be of comparable or superior sensory quality (i.e., overall sensory properties—taste, aroma, flavor, texture, and mouthfeel—that contribute to consumer satisfaction).

This review provides a comprehensive analysis of the physicochemical properties of apple varieties, their impact on sensory properties, and their changes during cider production, focusing on providing insights into the potential use of RFAs in novel cider production. Where information on RFAs is lacking, reference will be made to white‐fleshed apples (WFAs). Throughout this review, sensory changes are discussed within each relevant section, along with a dedicated section reporting the sensory properties of RFAs.

## 2 PHYSICOCHEMICAL PROPERTIES OF RFAs

The physicochemical quality of apples comprises characteristics such as color (skin/flesh), total soluble solids (TSS), titratable acidity (TA), texture, and phenolic composition (Jha et al., 2012). These traits serve as ripeness indicators, ensuring apples and apple products meet quality standards (Grabska et al., 2023). A meta‐analysis of over 800 apple varieties identified four key cider‐related parameters (TSS, TA, pH, and phenolics) (VanderWeide et al., 2022). It recommends blending apple juice to obtain a pH of <3.8 (to limit microbial spoilage), TA of 0.5%, and tannin level of 0.2% (Beech, 1972; Beech & Carr, 1977; Kumar et al., 2021; Valois et al., 2015). A review of 1774 studies reported TSS values in apples ranging from 5.31 to 21.60°Brix, with a mean of 12.44°Brix (VanderWeide et al., 2022). The significance of these parameters in cider production is explored in the following sections.

Furthermore, the apple variety has a substantial impact on the physicochemical quality of the fruit (Drkenda et al., 2021; Nicolini et al., 2018; Piagentini & Pirovani, 2017), apple juice (Fuleki et al., 1994; Tian et al., 2018), and cider (Keller et al., 2004; Riekstina‐Dolge et al., 2012). Due to natural variations in cider quality depending on the apple variety, the cider‐making process and adjuncts may need to be adapted. However, to understand which adjustments are needed to create a desirable end product, a comprehensive assessment of the apple/juice quality parameters (i.e., factors such as pH, sugar content, acidity, phenolic composition, and ripeness) and corresponding cider quality (i.e., the sensory balance of taste, aroma, flavor, mouthfeel, and overall complexity) should be done. Table 1 provides a summary of key component classes, specific compounds, their studied matrices, and their associated sensory impacts. This offers insights into the physicochemical properties’ role in shaping cider quality.

**TABLE 1:** Potential impact of key physicochemical properties on cider quality (sensory balance of taste, aroma, flavor, mouthfeel, and overall complexity).

Component class	Specific compounds	Studied matrix	Potential sensory impact on cider quality	Reference(s)
Sugars	Glucose	Apple juice	Contributes to sweet taste and product balance (sugar/acid ratio)	Plotkowski & Cline, 2021
	Fructose	Apple juice	Contributes to sweet taste and product balance (sugar/acid ratio)—highest sweetening index	Lončarić et al., 2023
	Sucrose	Apples	Contributes to sweet taste and balance (sugar/acid ratio)—less intense sweetness compared to glucose and fructose	Aprea et al., 2017
	Sorbitol	Apples	Affects smoothness, sweetness, and complexity—lowest sweetening index	Aprea et al., 2017
Organic acids	Citric acid	Wine, cider, apples	Contributes to acidic taste and promotes the perception of freshness Reduces browning Preserves flavors	Chen et al., 2016; Chidi et al., 2018; Los et al., 2017
	Malic acid	Apples (including RFA), ciders	Contributes to tart flavor (i.e., sharp and sour)	Bars‐Cortina et al., 2017; Cole et al., 2022
	Lactic acid	Wine, ciders	Creates a smoother and more refined taste (deacidification—reduces the sour taste of malic acid)	Sánchez et al., 2014; Sumby et al., 2010
	Succinic acid	Wine	Described as contributing a sour taste along with salty and bitter tastes	Coulter et al., 2004
	Quinic acid	Apples	Impacts astringency and plays an important role in sweet taste	Róth et al., 2007
Phenolic compounds	Flavan‐3‐ols	Apples, ciders	Contribute to bitterness, astringency, and mouthfeel	Lea & Arnold, 1978; Thompson‐Witrick et al., 2014
	Phenolic acid compounds	Apples (including RFA), ciders	Precursors of volatile compounds contributing to cider aroma Contribute to bitterness and astringency	Alonso‐Salces et al., 2001; Lachowicz et al., 2019; Sanoner et al., 1999
	Flavonols	Apples (including RFA)	Play a role in color stability/intensification of color (copigmentation reactions)—red color (anthocyanin stability) in the case of RFA and yellow color in certain apple varieties	Al Daccache et al., 2020; Brouillard et al., 1993; Malec et al., 2014; Mazza & Velioglu, 1992
	Dihydrochalcones	Apples	Responsible for bittersweet taste of apples	Thompson‐Witrick et al., 2014
	Anthocyanins	Apples (including RFA), apple juice, cider	Responsible for the red color in apple peel and flesh (in the case of RFA)	Ju et al., 1995; Knebel et al., 2018; Zuo et al., 2019

*Note*: Where literature on apples, apple juice, and ciders was unavailable, research on other fermented fruit (e.g., wine) was used.

Abbreviation: RFA, red‐fleshed apples.

### 2.1 Sugar content

The major soluble sugars reported in apples and apple juices are sucrose (15%–45% in WFA, 30%–39% in RFA), fructose (45%–59% in WFA, 51%–67% in RFA), glucose (14%–41% in WFA, 18%–25% in RFA), and sorbitol (sugar alcohol; 4%–14% in WFA, 2%–6% in RFA) (Jing et al., 2020; Juhart et al., 2022; Ramos‐Aguilar et al., 2017; Stander et al., 2021), the quantities of which can differ depending on several environmental conditions. The composition of sugars in the fruit influences fermentation kinetics and outcomes. For example, sucrose must first be hydrolyzed into glucose and fructose before fermentation can proceed (Chen et al., 2012). Since the latter sugars are readily metabolized, their presence can accelerate fermentation rates, as demonstrated in the kinetics model reported by Wang et al. (2004). The TSS (expressed in degrees Brix, °Brix) represents the concentration of dissolved substances in apples and their derived products (Merwin et al., 2008). Soluble sugars constitute the largest portion of TSS, making TSS measurements a reliable indicator of sugar content (Yang et al., 2021).

A North American and Canadian study found TSS content in 14 RFA varieties ranging from 9.4 to 17.5°Brix (Rupasinghe et al., 2010). More recent literature reported the TSS content of RFAs to be more consistent between varieties, ranging from 14.4°Brix (Contessa & Botta, 2016) to 14.9°Brix (Juhart et al., 2022). This may be related to the fact that almost all RFA varieties originate from the same crab apple (*Malus domestica* × *Niedzwetzkyana*). Alternatively, the small range may be due to the limited data available on RFAs. The TSS of the apple juice intended for cider is essential for fermentation, influencing both yeast metabolism (Section 4.7.1) and the sensory quality of the final product (Al Daccache et al., 2020; Călugăr et al., 2021; Wang et al., 2004). The sugar first serves as a substrate for the yeast, which ultimately converts sugar into ethanol and carbon dioxide (CO_2_) by alcoholic fermentation (Călugăr et al., 2021). In a well‐managed fermentation, most of the sugars are converted into ethanol, with only a small percentage forming byproducts (Lea & Piggot, 1996). Depending on the desired style (dry: <0.4% residual sugar; medium‐dry: 0.4%–0.9% residual sugar; medium: 0.9%–2.0% residual sugar; medium‐sweet: 2.0%–4.0% residual sugar; sweet: >4.0% residual sugar), fermentation can be halted at specific points to achieve the target residual sugar content (Beer Judge Certification Program, 2015). Naturally, the residual sugar after fermentation contributes to the sweetness of cider (Plotkowski & Cline, 2021) (Section 3.2; Table 1).

Given the importance of sugar for the cider fermentation process, growers breeding novel RFAs for cider production should focus on selecting high‐quality apples with a high TSS content (>12°Brix) and consider the sugar composition of the raw material (Aprea et al., 2012; Zhang et al., 2023). However, breeding apples for higher sugar content may result in unpredictable changes to other properties (e.g., flavor and sugar/acid balance). Another major consideration would be the ripeness and storage time, relating to starch degradation, which may affect the TSS content of the juice and/or reconstituted concentrate used for cider production. Understanding the sugar compositions in RFA varieties helps cider makers manage fermentation dynamics and flavor development, allowing for adjustments in fermentation conditions to achieve the desired outcomes.

### 2.2 Organic acids

TA is an indication of the sourness of the apples, their juice, and the final ciders (Moulton & Zimmerman, 2010). The two main functions of acids in cider making, similar to sugar content, are influencing fermentation (related to pH) and affecting the taste of the finished cider (related to TA) (Plotkowski & Cline, 2021) (Table 1). Numerous factors influence the concentration of organic acids in apples, including apple variety, ripeness, season, and climatic variations (Lea & Piggot, 1996). Apples contain nine to 12 organic acids in small fractions, but the main organic acids include malic (>80%), citric, oxalic, quinic, and succinic acids (Al Daccache et al., 2020; Del Campo et al., 2005; Lachowicz et al., 2019; Lea & Piggot, 1996). While lactic acid is not naturally found in apples, it is produced during malolactic fermentation (MLF), where it may contribute to the deacidification or the sensory complexity of ciders (Section 4; Table 1).

A study assessing the TA of 14 RFA varieties noted a large range of 0.31%–1.30% (Rupasinghe et al., 2010). Previous research found that the malic acid concentration in RFAs was higher (*p* < .05; 59.33–91.17 mg/kg flesh) than in WFAs (18.34–33.30 mg/kg flesh), except in *Granny Smith* apples, which had a similar (*p* > .05; 63.18 mg/kg flesh) acid content (Bars‐Cortina et al., 2017). However, the higher content of malic acid in RFAs could reduce consumer acceptance due to its contribution to tartness (i.e., a sharp and sour taste) (Bars‐Cortina et al., 2017) (Section 3.2; Table 1). Apart from malic acid, other minor organic acids (e.g., citric acid and quinic acid) have been identified in RFAs (Bars‐Cortina et al., 2017; Zhang et al., 2010). The contribution of these acids to the sensory properties of apples and ciders is summarized in Table 1.

The content of citric acid was also significantly higher (*p* < .05) in the *Baya Marisa* RFA variety (898 mg/kg FW) compared to *Golden Delicious* (570 mg/kg FW) (Juhart et al., 2022). However, a study by Bureau et al. (2012) found much lower citric acid values (0–180 mg/kg FW) in their study investigating organic acids in different apple varieties (*Golden Delicious*, *Fuji*, *Gala*, *Granny Smith*, Canada, *Chantecler*, and *Pink Lady*) with different techniques of sample preparation. Similar low values were reported in WFAs and RFAs in a study by Contessa & Botta (2016). However, differences in reported citric acid content may simply be due to genetic variation between apple varieties, variances in sample preparation methods, or variations in the analytical techniques used (Juhart et al., 2022). Nevertheless, citric acid even at low concentrations (0.5%–1% by weight) is essential in preserving fruit flavor and preventing browning (Chen et al., 2016; Jing et al., 2016; Rahman et al., 2010). Its presence noted in RFAs could play a crucial role in preserving the quality and flavor of juices and ciders produced from these apples.

In addition, the pH of the juice is another critical factor influencing microbial stability and fermentation. The pH of a juice influences the survival of yeast, beneficial bacteria (e.g., *Leuconostoc* spp.), and spoilage microorganisms (e.g., *Pediococcus* and *Lactobacillus* spp.), as well as the formation of H_2_S, biogenic amines, and volatile acids (Du Toit & Pretorius, 2000). In apple juice, a pH of >3.8 could result in microbial growth and the development of off‐flavors during cider fermentation (Kumar et al., 2021). Therefore, some varieties are at a higher risk of contamination by bacteria (e.g., *Lactobacillus* spp., *Pediococcus* spp., and *Acetobacter*) and wild yeasts (e.g., *Saccharomyces bayanus*, *Saccharomyces cerevisiae*, *Kloeckera apiculata*, *Metschnikowia pulcherrima*, and related spp.) during fermentation and after bottling (Kumar et al., 2021; Merwin et al., 2008; Nicolini et al., 2018; Valois et al., 2015).

Studies have shown minimal intra‐fruit variability for TSS in RFAs (Section 2.1), but similar data for organic acids are lacking. While RFAs may have suitable acidity levels for flavor balance and microbial stability, further research is needed to fully understand their application in cider production. The organic acid levels in RFAs suitable for fresh consumption, juice, or cider remain undefined. This underscores the complexity of acidity in cider taste (Section 3.2) and the need to explore its impact on consumer preferences.

### 2.3 Major phenolic compounds in apples

The phenolic compounds in apples are synthesized during growth until the full maturity stage (Zhang et al., 2010) and dispersed throughout its tissues (epicarp/exocarp/skin, mesocarp/flesh, endocarp/core, and seeds/pips) (Alberti et al., 2017; Łata et al., 2009). However, the distribution of phenolic compounds also differs based on the apple variety (Alberti et al., 2017; Feng et al., 2021; Kalinowska et al., 2014; Łata et al., 2009).

The apple peel is only 6%–8% of the fruit weight, but a study on WFAs demonstrated that the peel contains substantially higher concentrations of catechin (2.21 vs. 0.88 mg/kg dry weight [DW]), epicatechin (2.60 vs. 0.75 mg/kg DW), rutin (5.43 vs. 0.82 mg/kg DW), and phloridzin (1.55 vs. 0.27 mg/kg DW) than the flesh, while chlorogenic acid was found to be more abundant in the flesh (0.95 vs. 1.13 mg/kg DW) (Łata et al., 2009). Studies by Tsao et al. (2003) and Alberti et al. (2017) confirmed that anthocyanins and flavonols are almost exclusively found in the peel of WFAs. Similarly, RFAs also showed a higher total phenolic content in the peel than in the flesh across all developmental stages (young stage: 3234–9082 mg gallic acid equivalents [GAE]/kg in peel vs. 2523–5346 mg GAE/kg in flesh; development stage: 2637–5460 mg GAE/kg in peel vs. 1127–2397 mg GAE/kg in flesh; mature stage: 1240–3334 mg GAE/kg in peel vs. 328–2284 mg GAE/kg in flesh) (Zhang et al., 2020). Another study investigating the phenolic composition of Chinese RFAs found that the total phenolic compounds in the peel were significantly higher (*p* < .05; 5430 vs. 3087 mg GAE/kg) compared to the flesh (Katiyo et al., 2018). The polyphenols of apples are critical for cider quality (Sanoner et al., 1999). Therefore, it is vital to understand the types and distribution of phenolic compounds in apple varieties to understand their functionality (Lee et al., 2017) (Sections 2.3.1–2.3.5).

Vrhovsek et al. (2004) reported the polyphenol content of eight of the most extensively grown dessert apple varieties in the South and West of Europe in increasing order of phenolic concentration (*Fuji*, *Braeburn*, *Royal Gala*, *Golden Delicious*, *Morgenduft*, *Granny Smith*, *Red Delicious*, and *Renetta*). Flavan‐3‐ols (monomeric flavan‐3‐ols and procyanidins [oligomeric flavan‐3‐ols]) contributed the majority (71%–90%), followed by hydroxycinnamic acids (4%–18%), flavonols (1%–11%), dihydrochalcones (2%–6%), and anthocyanins (1%–3%; in red‐skinned apples) (Vrhovsek et al., 2004). Wojdyło et al. (2008) in their study of 67 old and new varieties grown in Western Europe showed similar results. While RFAs such as *Baya Marisa*, *Weirouge*, *Roberts Crab*, and *Hongrouguo* generally exhibit higher levels of total polyphenols, total flavan‐3‐ols, total anthocyanins, and total dihydrochalcones compared to WFAs such as *Golden Delicious* and *Gale Gala* (Juhart et al., 2022; Sadilova et al., 2006; Wang et al., 2015), this trend is not absolute. Juhart et al. (2022) found that certain WFAs, including *Golden Delicious*, contained higher flavan‐3‐ol and hydroxycinnamic acid levels than some RFAs. This highlights an area of overlap, indicating that while RFAs tend to have a higher phenolic content overall, variability exists depending on the specific varieties studied. Therefore, comparisons should consider variety‐specific differences rather than assuming a strict division between WFAs and RFAs.

**FIGURE 1:**
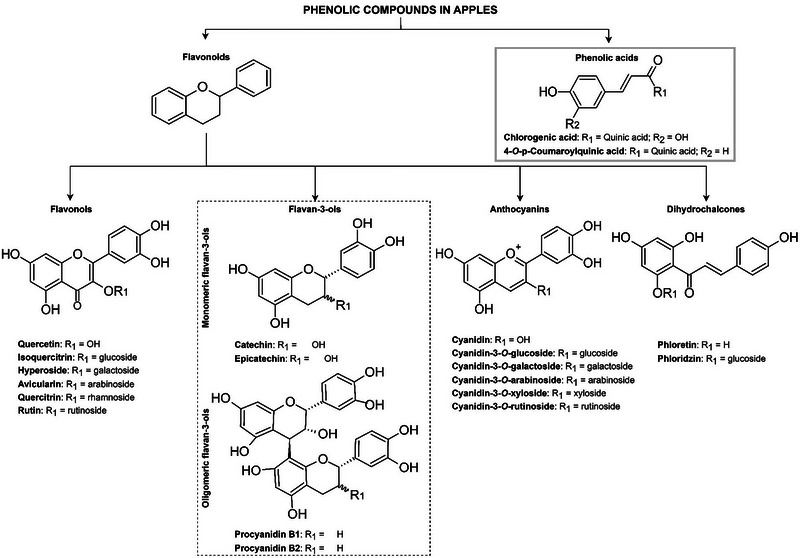
Major phenolic compounds and their structures commonly found in all apple categories (i.e., culinary apples, dessert apples, cider apples, and red‐fleshed apples).

An optimal concentration of phenolic compounds in cider apple juice has not been defined, and the absence of a standardized commercial protocol for cider production further complicates the establishment of such a threshold (Alexander et al., 2019). This challenge is particularly pronounced for RFAs, where phenolic composition is elevated and can vary greatly among varieties. However, the selection of RFAs with naturally higher phenolic content presents an opportunity for cider producers to enhance the sensory complexity of their products (Marks et al., 2007a; Thompson‐Witrick et al., 2014). In the forthcoming sections of this review, specific attention is directed toward examining the phenolic groups that prominently influence cider quality: flavan‐3‐ols, phenolic acid compounds, flavonols, dihydrochalcones, and anthocyanins (Figure 1).

#### 2.3.1 Flavan‐3‐ols

Flavan‐3‐ols are a significant class of phenolic compounds in apples (Henry‐Kirk et al., 2012). These compounds comprise monomeric (catechin and epicatechin), oligomeric (procyanidins with two to four monomer subunits), and polymeric (procyanidins with more than four monomer subunits) forms (Wojdyło et al., 2008) (Figure 1). The flavan‐3‐ol polymers (condensed tannins) are defined as polyphenols that bind to and precipitate proteins and other organic compounds (Alonso‐Salces et al., 2004; Karl & Peck, 2022; Lea & Arnold, 1978; Siebert et al., 1996). They play a critical role in the sensory quality (specifically bitterness and astringency) of apples and apple products, such as ciders.

Despite different analytical methods across studies, procyanidins are the most abundant class of polyphenols (69%–87%) in commercial dessert apple varieties (Guyot et al., 2002; Lončarić et al., 2020; Sanoner et al., 1999; Vrhovsek et al., 2004). Various studies showed a substantially higher flavan‐3‐ol content in WFAs compared to RFAs in both the flesh and skin (Bars‐Cortina et al., 2017; Juhart et al., 2022). This was postulated to be due to a competitive interaction between leucoanthocyanidins (precursor to flavan‐3‐ols and anthocyanins) and two distinct enzymes (anthocyanidin synthase and anthocyanidin reductase) (Henry‐Kirk et al., 2012; Yuste et al., 2022). However, Espley et al. (2013) found that red‐fleshed *Royal Gala* apples (a transgenic apple line) did not have a lower flavan‐3‐ol content due to their high anthocyanin expression (values approaching that of dark plums, 251 mg/kg FW) (Koponen et al., 2007).

Recent public interest in the health‐promoting properties of tannins offers marketing opportunities for nonalcoholic processed products made from tannin‐rich RFA varieties (Bars‐Cortina et al., 2017). Since procyanidins are important for the quality of apples and apple products, their precise quantification is extremely important (Guyot et al., 2001). Due to the lack of standards, both spectrophotometric and chromatographic techniques rely on estimates (Guerrero‐Hurtado et al., 2023; Watrelot & Norton, 2020; Wilhelmy et al., 2021). Obtaining standards for quantification becomes more challenging with complex tannins due to isomerism and polymerization (Schofield et al., 2001). Therefore, new and robust analytical techniques or optimization of existing methods is needed to accurately identify and quantify tannin composition.

#### 2.3.2 Phenolic acid compounds

Phenolic acid compounds are the second‐largest phenolic class in apples (Da Silva et al., 2019) and the most abundant phenolic class in fruits (Pereira et al., 2009; Shi et al., 2022). These compounds comprise hydroxybenzoic acids (C6–C1, benzene ring with one carboxylic acid group) and hydroxycinnamic acids (C6–C3, benzene ring with a three‐carbon chain attached) that are typically present as esters, amides, and glycosides (Lee et al., 2017; Pereira et al., 2009). Of the two groups, hydroxycinnamic acids are more frequently quantified in apples, with chlorogenic acid (5‐*O*‐caffeoylquinic acid) being the most abundant in apples, apple juices, and ciders, followed by 4‐*O*‐*p*‐coumaroylquinic acid (Al Daccache et al., 2020; Da Silva et al., 2019; Kalinowska et al., 2014; Tsao et al., 2003). Notably, hydroxycinnamic acids are precursors of volatile compounds contributing to cider aroma (Alonso‐Salces et al., 2001; Sanoner et al., 1999) (Table 1). Gaining an understanding of the range and distribution of phenolic acid compounds in apples is essential to comprehending their functionality in the raw material and cider, as well as the final cider quality.

Phenolic acid compounds are the predominant phenolic compounds in RFAs, representing more than 50% of the total polyphenols (Bars‐Cortina et al., 2017; Lee et al., 2017; Wang et al., 2015; Yuste et al., 2020). Phenolic acid compounds, specifically chlorogenic acid, play a crucial role in determining the flavor profile of ciders as they contribute to astringency and bitterness (Lachowicz et al., 2019). Bars‐Cortina et al. (2017) found that chlorogenic acid concentrations in the flesh and peel of RFAs (*Redlove Era 107/06*, *117/06*, *119/06*, and *RS‐1*) were similar to those in WFAs (*Brookfield Gala*, *Zhen Aztec Fuji*, *Golden Smoothee*, and *Inored*), except for *Granny Smith* and *Story* with lower (*p* < .05) concentrations. However, other minor phenolic acid compounds (i.e., protocatechuic acid and vanillic acid) were all reported at higher (*p* < .05) concentrations in RFAs than in WFAs (Bars‐Cortina et al., 2017).

Understanding the phenolic acid compound composition in apples, particularly hydroxycinnamic acids, is crucial for optimizing the sensory quality and stability of ciders. Since these compounds serve as precursors to volatile compounds that shape cider aroma, their balanced presence is key to developing desirable flavor profiles. The higher levels of minor phenolic acid compounds in RFAs, as compared to WFAs, could provide unique opportunities to craft ciders with distinctive sensory attributes that cater to emerging consumer preferences for more complex and bold flavor experiences.

#### 2.3.3 Flavonols

Flavonols are a flavonoid subclass that contributes to the yellow color of certain apple varieties (Al Daccache et al., 2020; Malec et al., 2014). In apples, the main flavonols are glycosides (galactose, glucose, arabinose, xylose, and rhamnose) of quercetin, kaempferol, and myricetin (Kim et al., 2003), with quercetin glycosides being most abundant (Alonso‐Salces et al., 2004; Spanos & Wrolstad, 1992). Unfortunately, these compounds represent less than 0.5%–1% of the total phenolic compounds in apples and are poorly extractable (Guyot et al., 2003; Malec et al., 2014; Marks et al., 2007b). Ciders have significantly lower flavonol glycoside concentrations than their corresponding juice (*p* < .05; 44.4 mg/L in juice vs. 29.1 mg/L and 30.6 mg/L in ciders), likely due to poor retention during fermentation, acid hydrolysis of glycosides, and/or oxidation (He et al., 2022). Furthermore, some conjugates are cleaved during cider making by pectolytic enzymes (Section 4.3) with glycosidase activity, which releases the aglycones (He et al., 2022; Madrera et al., 2006; Oszmiański et al., 2007). However, the low flavonol concentrations in juices and ciders should not overshadow their significance as they play a vital role in enhancing antioxidant capacity, color stability, and sensory qualities in RFAs and their derived products.

In a study investigating the phytochemical profiles of RFAs versus traditional and new WFAs, the main flavonols detected in the peel and flesh of all nine apple varieties were quercetin derivatives (Bars‐Cortina et al., 2017). While Bars‐Cortina et al. (2017) found no difference in the concentration of flavonols between RFAs and WFAs, another study found that RFA varieties (*Xiahongrou*, *Hongrouguo*, and *No. 1 Hongrouguo*) yielded lower flavonol concentrations in their peel (182–653 mg/kg FW) but higher flavonol content in their flesh (179–473 mg/kg FW) compared to *Golden Delicious* apples (1285 mg/kg FW in peel and 129 mg/kg FW in flesh) (Wang et al., 2015). In *Gale Gala* and *Golden Delicious* apples, flavonols accounted for 41% and 56% of total phenolics in the peel, respectively, while only representing 12.6%–29.4% of total phenolics in the peel of four RFAs (Wang et al., 2015). A similar tendency was observed in the flesh of the apples (Wang et al., 2015). This disparity between WFAs and RFAs may be related to the higher anthocyanin concentration in RFAs, with values ranging from 14 to 301 mg/kg FW in WFAs compared to 295–1758 mg/kg FW in RFAs in the peel, and no anthocyanin detected in WFAs versus 12–560 mg/kg FW in RFAs in the flesh (Wang et al., 2015). Importantly, dihydroflavonol 4‐reductase, a crucial enzyme in anthocyanin synthesis, competes directly with flavonol synthase for the same substrates, dihydrokaempferol and dihydroquercetin (Martens et al., 2002). The dihydroflavonols are the branching point in the pathway for anthocyanin and flavonol production. Consequently, increased anthocyanin concentration could reduce the flavonol concentration in RFAs, potentially leading to ciders with lower antioxidant capacity, color stability, and diminished flavor complexity.

#### 2.3.4 Dihydrochalcones

Apples are the major contributor of dihydrochalcones in the diet (Bars‐Cortina et al., 2018; Stompor et al., 2019; Yuste et al., 2022), with only minor amounts found in exotic fruits such as guava and starfruit (Rojas‐Garbanzo et al., 2017). Despite low concentrations of dihydrochalcones in apples, their exclusivity allows their use as a biomarker for the presence of apple juice in other fruit juices (Alberti et al., 2017). Dihydrochalcones can be present as aglycons or glycosides (e.g., xyloglucoside and glucoside) or substituted by different groups (e.g., *C*‐benzylated) (Wojdyło et al., 2008). The main dihydrochalcones in WFAs and RFAs are phloretin‐2′‐*O*‐xyloglucoside (PXG) and phloridzin (phloretin‐2′‐*O*‐glucoside) (Alonso‐Salces et al., 2001; Bars‐Cortina et al., 2017; He et al., 2022; Juhart et al., 2022; Lommen et al., 2000; Zardo et al., 2013). Phloridzin, the major phenolic compound responsible for the bittersweet taste of apples (Table 1), was found in the highest concentration in the flesh (0.0–63.5 mg/kg FW) and peel (0.0–414.0 mg/kg FW) of 20 cider, processing, and dessert apple varieties, while phloretin derivatives were present only in trace amounts (<0.01 mg/kg FW) (Thompson‐Witrick et al., 2014).

Recent publications reported that RFAs have higher dihydrochalcone concentrations in their peels than conventional WFAs (185.3 vs. 56.2 mg/kg FW in the peel [Juhart et al., 2022]; 375 vs. 176.0 mg/kg FW in the peel [Yuste et al., 2022]). A similar trend in these studies was observed in the flesh of the apples, with the only difference being lower concentrations of dihydrochalcones across all apple varieties. This difference in dihydrochalcone distribution between RFAs and WFAs was attributed to chalcone synthase activity, which increases with anthocyanin production in the skin of RFAs during fruit ripening. Furthermore, the scab‐resistant nature of some RFA varieties (e.g., *Baya Marisa*) is linked to the increased levels of phloridzin, a functional antioxidant that may reduce oxidative stress in the fruit (Gosch et al., 2009; Juhart et al., 2022; Schmitzer et al., 2023).

In the case of RFAs, the higher concentrations of dihydrochalcones can enhance the bitterness and complexity of ciders. They also contribute to the antioxidant potential of apple products, highlighting the multifaceted importance of measuring these compounds to ensure the overall quality and characteristics.

#### 2.3.5 Anthocyanins

The pigments responsible for the red color in apple peel and flesh (for RFAs) are anthocyanins (Iglesias et al., 2012). Anthocyanidins, aglycons of anthocyanins, are usually glycosylated with glucose, rhamnose, galactose, xylose, or arabinose (Chen et al., 2021; Wang et al., 2021) (Figure 1). Fruit color is mainly determined by these different anthocyanins and shows varying hues (e.g., red, purple, and blue) depending on the anthocyanin composition and pH in the cells (Alappat & Alappat, 2020; Chen et al., 2021) (Table 1). Furthermore, colorless phenolic compounds can facilitate the intensification of apple color through copigmentation (Brouillard et al., 1993; Mazza & Velioglu, 1992).

Anthocyanins are often present in very low concentrations (undetectable to 30 mg/kg DW or 208 mg/kg FW) in dessert apples with green/yellow (*Ozark Golden*, *Mutsu*, and *Golden Delicious*), blush (*Ligol* and *Delbarestivale*), or red skin (*Topaz*, *Fuji*, *Shampion*, *Red Delicious*, *Empire*, *Ida Red*, *McIntosh*, *Northern Spy*, *Cortland*, and *Rubinola*) (Juhart et al., 2022; Tsao et al., 2003; Wang et al., 2015; Wojdyło et al., 2008). The anthocyanins of red‐skinned apples are typically restricted to a few hypodermal and epidermal cell layers, and the flesh does not usually contain anthocyanins (Juhart et al., 2022; Van Nocker et al., 2012; Wang et al., 2015).

In several studies, with RFAs including *Weirouge* (Sadilova et al., 2006) or *Niedzwiedzkyana* (Rupasinghe et al., 2010), cyanidin‐3‐*O*‐galactoside (idaein) has been identified as the most abundant anthocyanin (∼95% of the total anthocyanin content) (Bars‐Cortina et al., 2017; Mazza & Velioglu, 1992; Sadilova et al., 2006; Stander et al., 2021; Vrhovsek et al., 2004; Würdig et al., 2014). Apart from idaein, cyanidin‐3‐*O*‐xyloside and cyanidin‐3‐*O*‐arabinoside have also been detected (Li et al., 2020a; Sadilova et al., 2006). Bars‐Cortina et al. (2017) found that the total anthocyanin content in the flesh of four RFAs (*Redlove Era 107/06*, *117/06*, *119/06*, and *RS‐*1; 9.34–49.3 mg/kg) was much higher compared to WFAs (*Brookfield Gala*, *Zhen Aztec Fuji*, *Golden Smoothee*, *Granny Smith*, and *Story* [*Inored*]; 0.60–1.23 mg/kg). Multiple studies have shown similar results (Bars‐Cortina et al., 2018; Contessa & Botta, 2016; Espley et al., 2013; Stander et al., 2021; Van Nocker & Gottschalk, 2017; Yuste et al., 2022). Furthermore, the varying anthocyanin levels among RFA varieties indicate differences in the conversion from flavan‐3‐ols to anthocyanins, and these anthocyanin compounds also undergo degradation during maturation (Wang et al., 2015).

Although anthocyanins provide a vibrant red hue, numerous health benefits in apples, and protection against oxidative damage (Juhart et al., 2023; Rupasinghe et al., 2010; Van Nocker et al., 2012), higher anthocyanin concentrations have been linked to fruit browning (localized in the fruit cortex) (Espley et al., 2013). However, the exact anthocyanin threshold at which browning occurs in RFAs has not been clearly defined in the literature. Flesh browning may result from internal membrane instability at low temperatures (0–5°C) during storage, leading to anthocyanin leakage from the vacuole and interaction with cellular enzymes such as polyphenol oxidase (PPO) (Espley et al., 2013; Saba & Watkins, 2020; Sidhu et al., 2023). Nevertheless, the high acidity reported in the fruit (1190 mg malic acid/100 g edible portion) may help counteract this effect by stabilizing the anthocyanin color (Sadilova et al., 2006).

There remains a noticeable gap in understanding the changes in anthocyanins during RFA juice‐ and cider processing and storage. As cider production involves various stages that could alter the anthocyanin composition (Section 4), a more focused investigation into these changes is warranted. When selecting apples with an intense red peel and flesh color, breeders would want to know if and which other polyphenols are altered during processing. This will allow stakeholders to understand the potential use of RFAs for cider production.

## 3 SENSORY PROPERTIES OF RFAs

Across the globe, apple breeders strive to develop apple varieties with improved appearance, taste, flavor, and flesh texture (Yuste et al., 2022). Physicochemical properties are key quality indicators during apple breeding programs (Kumar et al., 2022; Volz et al., 2009) and are equally important in apple juice manufacturing (Malec et al., 2014; Park et al., 2020; Thompson‐Witrick et al., 2014) and cider production (Nicolini et al., 2018; Sanoner et al., 1999). However, processing alters these properties, affecting sensory properties and consumer acceptance of the final product.

Investigating RFA sensory properties across different stages requires evaluation methods tailored to specific goals. Different methods capture key sensory attributes (i.e., color, aroma, flavor, taste, and texture/mouthfeel) essential for apple selection and cider quality. The method depends on the study objectives, whether it is understanding consumer preferences during breeding and selection, optimizing fermentation processes in cider production, or ensuring consistent quality during storage. Each sensory evaluation approach has benefits and limitations, so breeders, cider makers, and researchers must choose methods aligning with objectives, resources, and RFA properties. This ensures a thorough understanding of RFAs’ role in crafting high‐quality, distinctive ciders.

Conventional breeding practices for apples often rely on the expertise of a few breeders to identify the most promising selections based on their sensory properties, but this method is very subjective (Hampson et al., 2000). This is particularly relevant for RFAs, where breeding programs must balance horticultural aspects and disease resistance with desirable sensory attributes to optimize their use in cider production (Bulley et al., 2007). Breeders often use simple sensory assessments, such as phenotypic selection, to evaluate traits such as flavor, aroma, texture, and juiciness in apples and RFAs (Migicovsky et al., 2016; Sestras & Sestras, 2023; Wang et al., 2018). Furthermore, visual assessments, using color scales or digital imaging, are employed to evaluate external characteristics such as skin and flesh color (Bouillon et al., 2024).

While simple sensory assessments can help identify promising varieties, they are subjective, do not provide insights into the broader sensory profile of new apple varieties, and can lead to inconsistent outcomes. To improve consistency and provide a deeper understanding of the sensory potential of RFAs for cider production, incorporating more standardized and advanced sensory methods such as analytical and hedonic methods is recommended. A detailed discussion of the various sensory methods for evaluating RFA varieties and their products is beyond the scope of this review. Instead, the following sections will focus on the fundamental sensory properties of RFAs.

### 3.1 Appearance

The importance of the appearance of a food product and the individual factors that contribute to the overall perception of the product based on appearance has long been known (Hutchings, 1977). Apple varieties display a range of colors, from red, green, and yellow to bicolored due to variations in the type and amount of pigments in the skin (Drogoudi et al., 2008; Jing et al., 2020). As discussed in Section 2.3.5, anthocyanins are the pigments in apple skin and/or flesh that are responsible for the red color (Iglesias et al., 2012; Jing et al., 2020; Mazza & Velioglu, 1992; Wang et al., 2021). A study by Drkenda et al. (2021) concluded that apples with red skin color were more accepted by consumers than those with yellow skin color. Similar results were found in a previous study by Bars‐Cortina et al. (2018). However, a study on different Korean apple varieties found that although consumers (*n* = 100) prefer red‐skinned apples (i.e., external color), they did not prefer apples with red flesh (i.e., internal color) (Kim et al., 2023). Since the study focused exclusively on a limited demographic, caution should be exercised when attempting to generalize the findings to a broader global perspective. Furthermore, the novelty of RFA varieties could mean that consumers need more time and exposure to adapt.

Much work remains in acquainting consumers with the novelty of RFAs and their appearance. One potentially effective approach is leveraging apple‐based products such as ciders. RFA ciders, therefore, present considerable potential provided that efforts ensure color stability, especially during cider processing, where phenolic compounds responsible for color and color stability undergo significant changes (Sanoner et al., 1999) (Section 4).

### 3.2 Palate attributes—Taste

Like skin and flesh color, apple taste development is a complex process influenced by environmental factors (Hecke et al., 2006; Jing et al., 2020). Sugar and acid contents have a notable influence on the sensory properties of apples and apple products, as well as consumer acceptance of these products (Ackermann et al., 1992; Bars‐Cortina et al., 2017; Espley et al., 2013; Etienne et al., 2013; Feliciano et al., 2010; Fuleki et al., 1994; Juhart et al., 2022).

While acidity plays a crucial role in flavor balance, excessive levels can lead to a decline in consumer preference. McCracken et al. (1994) found that apples with moderate malic acid concentrations (1500–1800 mg/kg juice), such as *Hawaii* and *Red Delicious*, were well‐received, whereas those with significantly higher levels (*p* < .05, 31,200–31,600 mg/kg juice) were less preferred. However, similar consumer preference data have not been established for RFAs, making it unclear whether their naturally higher malic acid content, such as the 9685 mg/kg reported in *Baya Marisa* (Juhart et al., 2022), could negatively impact the acceptance of RFA fruit or cider.

While a higher acid content is crucial for a fresh taste and balancing sugar levels in apples (Wicklund et al., 2020), the sensory experience of fruits, juices, and ciders depends on the balance between sweet and sour tastes. The sugar‐to‐acid ratio (TSS/TA, which influences the perceived sweetness and overall flavor balance of apples and apple products) plays a crucial role in determining cider quality and palatability (Wu et al., 2007). Furthermore, the TSS/TA value is important in determining whether the apple fruit is suitable for direct consumption or processing. A higher TSS/TA ratio generally indicates a sweeter taste, which is more appealing to consumers. Apple varieties with TSS/TA values ≥40 have been recommended for fresh consumption, while those with lower ratios (<40) are often better suited for juice or cider production (Mureșan et al., 2022; Wu et al., 2007; Zhang et al., 2023). For instance, Zhang et al. (2023) reported that *Honglu* apples had a TSS/TA ratio of 70.71, making them suitable for fresh consumption, whereas *Miqila* apples had a ratio of 38.21, indicating a more tart flavor, which may be better suited for processing.

While organic acids contribute to astringency and bitterness, phenolic compounds are the primary contributors to these properties in RFAs (Lawless et al., 1996; Marks et al., 2007b). Although pronounced bitterness may be undesirable for fresh consumption, it adds complexity and depth to cider flavor (Soares et al., 2013). The phenolic content and resulting sensory properties (often focusing on bitterness) of cider apples are the subject of ongoing research in European countries such as Spain (Alonso‐Salces et al., 2004, 2006), the United Kingdom (Lea & Arnold, 1978; Marks et al., 2007a), France (Guyot et al., 2003; Sanoner et al., 1999), and Poland (Wojdyło et al., 2008). Additionally, some work has been done in apple‐growing regions of the United States (Kelkar & Dolan, 2012; Merwin et al., 2008; Valois et al., 2015). High‐phenolic‐content cider apples, including RFAs, often exhibit increased bitterness, which can enhance the sensory appeal of ciders, especially in styles where bitterness is desired (Alonso‐Salces et al., 2004; Sanoner et al., 1999). However, specific sensory data quantifying the bitterness of RFAs in relation to their phenolic content are limited, highlighting the need for further research.

Apple taste, sweetness, acidity, and bitterness shape the sensory foundation of ciders. The balance of these tastes, influenced by variety and cultivation, defines the final cider taste profile. Cider makers can refine and manipulate these elements to craft unique RFA ciders.

### 3.3 Palate attributes—Aroma and flavor

Aroma is a key sensory property that determines the quality of food and fermented fruit beverages (Alberti et al., 2016). Identifying individual volatile compounds’ contributions to orthonasal and retronasal aroma is challenging (Pello‐Palma et al., 2016). Aroma is influenced by a product's qualitative and quantitative volatile compound composition (Liu et al., 2020) (Table 2) and affects fruit‐based product acceptability (El Hadi et al., 2013). While many fruits share similar aromatic compounds, RFAs offer a distinct aroma profile due to specific concentrations, combinations, and perception thresholds of their volatile compounds (Arcari et al., 2017; El Hadi et al., 2013). Although some studies have identified key volatile compounds contributing to the aroma of a limited number of RFAs (Červenčík et al., 2022; Chitarrini et al., 2021), further research is needed to fully characterize their profiles and development in cider making.

**TABLE 2:** Relative concentration of volatile compounds identified in ciders of different apple varieties by headspace solid‐phase microextraction coupled to gas chromatography–mass spectroscopy (HS‐SPME/GC–MS).

Common compound name (IUPAC nomenclature/alternative names)	Concentration in cider (mg/L)	Perception threshold (mg/L)	Aroma description	Reference for aroma description
Alcohols				
Propanol (propan‐1‐ol/*n*‐propanol)	0.002–0.005^a^, ^d^ 0.002–0.092^e^ 0.013–0.084^k^ 19.1–55.1^k^	9	Fermented, fruity, apple, pear Fresh, alcohol (ethanol), ripe fruit	Rosend, Kuldjärv, Rosenvald, et al., 2019 Li et al., 2020b
Isobutanol (2‐methylpropan‐1‐ol/i‐butanol)	21.7–85.8^k^	40^j^	Unpleasant, wine‐like, alcoholic, fruity	Červenčík et al., 2022
Butanol (butan‐1‐ol/*n*‐butanol)	0.011–0.015^a^, ^d^ 0.020–0.412^e^ 3.4–7.1^k^ 16.1–18.8^l^	150^j^	Balsamic Medicinal, alcohol	Rosend, Kuldjärv, Rosenvald, et al., 2019 Li et al., 2020b
Isoamyl alcohol (3‐methylbutan‐1‐ol/isopentyl alcohol)	118.7–226.0^k^ 77.2–97.1^l^	30	May contribute to an unpleasant flavor Alcohol, harsh, cheese Chocolate, herbal	Rous & Snow, 1983 Simonato et al., 2021 Wei et al., 2020
Hexanol (hexan‐1‐ol/*n*‐hexanol)	0.174–0.413^d^ 0.284–4.659^e^ 4.7–8.3^k^	8^h^ 80^j^	Fresh, green, apple undertone, apple pips‐like Green, pungent Floral Green, grass	Červenčík et al., 2022 Rosend, Kuldjärv, Rosenvald, et al., 2019 Kliks et al., 2021 Li et al., 2020b
2‐Phenyl ethanol (2‐phenylethan‐1‐ol/phenethyl alcohol)	0.0–0.599^e^ 9.21–435.8^k^ 8.0–15.0^l^	14^g^, ^j^ 10^h^	Floral, sweetish, rose, slight honey‐like Floral, rose Flowery, bread‐like Roses, pollen, perfume Rose, honey	Červenčík et al., 2022 Rosend, Kuldjärv, Rosenvald, et al., 2019 Kliks et al., 2021 Li et al., 2020b Simonato et al., 2021
Esters				
Ethyl acetate (ethyl acetate/ethyl ethanoate)—products of alcohols and acetic acid^b^	0.076–0.335^a^, ^d^ 0.010–0.714^e^ 59.1–126.1^k^	7^h^ 12.3^j^	Ethereal, solvent‐like, slight fruity undertones Pleasant, fruity Fruity, green Pineapple, fruity, balsamic Vinegar	Červenčík et al., 2022 Satora et al., 2008 Rosend, Kuldjärv, Rosenvald, et al., 2019 Li et al., 2020b Simonato et al., 2021
Ethyl butyrate (ethyl butanoate/butyric ether)—condensation of ethanol and butyric acid^b^	0.011–0.023^a^, ^d^ 0.003–0.151^e^	0.02^h^ 0.125^j^	Pineapple, cognac Fruity, sweet Apple‐like, sweet Strawberry, apple, banana Fruity, strawberry Sour fruit, strawberry, fruity	Rosend, Kuldjärv, Rosenvald, et al., 2019 Kliks et al., 2021 Červenčík et al., 2022 Li et al., 2020b Simonato et al., 2021 Tao & Li, 2009
Isoamyl acetate (3‐methylbutyl ethanoate/isopentyl acetate)—isoamyl alcohol and acetic acid^b^	0.004–0.253^e^ 0.6–2.1^k^	0.03^j^	Banana, pear Banana	Rosend, Kuldjärv, Rosenvald, et al., 2019 Simonato et al., 2021
Ethyl caproate (ethyl hexanoate/caproic acid ethyl ester)—hexanoic acid and ethanol^b^	0.003–0.003^f^ 0.010–0.255^e^	0.062^i^, ^j^	Fruity, pineapple, banana Fruity, green apple, floral, violet Fruity, green apple Fruity, sweet	Rosend, Kuldjärv, Rosenvald, et al., 2019 Ferreira et al., 2000 Simonato et al., 2021 Wei et al., 2020
Hexyl acetate (hexyl acetate/*n*‐hexyl ethanoate)—hexanol and acetic acid^b^	0.005–0.364^a^, ^d^ 0.001–0.166^e^	1.5^j^	Apple, fruity, floral, slightly bitter, apple pips‐like Fruity, green apple, banana Pleasant, fruity, pear, cherry Apple	Červenčík et al., 2022 Rosend, Kuldjärv, Rosenvald, et al., 2019 Li et al., 2020b Simonato et al., 2021
Ethyl lactate (ethyl 2‐hydroxypropanoate/lactic acid ethyl ester)—ethanol and lactic acid^b^	164.7–337.0^k^	154^j^	Fruity, strawberry, floral	Ferreira et al., 2000
Ethyl caprylate (ethyl octanoate)—octanoic acid and ethanol^b^	0.241–8.039^a^, ^d^	0.002^h^ 0.005^g^ 0.58^j^	Fruity, pineapple, pear, floral	Ferreira et al., 2000
Ethyl caprate (ethyl decanoate/decanoic acid ethyl ester)—capric acid and ethanol^b^	0.047–1.342^a^, ^d^ 0.293–0.380^f^	0.2^g^, ^j^	Waxy, fruity, apple, grape Waxy, fruity, rose	Rosend, Kuldjärv, Rosenvald, et al., 2019 Li et al., 2020b
Phenethyl acetate (2‐phenyl ethyl acetate/2‐phenethyl acetate)—acetic acid and phenethyl alcohol^b^	0.003–0.166^d^ 0.0–0.024^e^ 0.1^k^	0.25^j^	Fruity, sweet, floral Honey, rose Rose, pleasant, flowery Floral, rose	Červenčík et al., 2022 Rosend, Kuldjärv, Rosenvald, et al., 2019 Li et al., 2020b Simonato et al., 2021
Aldehydes and ketones				
Acetaldehyde (ethanal/acetic aldehyde)	0.009–0.015^a^, ^d^	0.5^h^, ^j^	Green apple, sour, metallic Pleasant, fruity (low concentrations) Green fruit (low concentrations), unpleasant (high concentrations) Fruity, pungent Yoghurt, alcoholic	Satora et al., 2008 Dos Santos et al., 2018 Wicklund et al., 2020 Simonato et al., 2021 Wei et al., 2020
Acetoin (3‐hydroxybutan‐2‐one/acetyl methyl carbinol)	9.5–21.7^m^	150^g^, ^j^	Buttery Off‐flavor	Jagtap & Bapat, 2015 El Hadi et al., 2013
Acids and fatty acids				
Acetic acid (ethanoic acid/ethylic acid)	0.011^d^	200^h^ 300^j^	Together with ethyl acetate = volatile acidity	Satora et al., 2008
Isobutyric acid^c^ (2‐methylpropanoic acid/isobutanoic acid)	2.5–4.1^k^	0.05^j^	Rancid Blue cheese, dirty	Williams & Carter, 1977 Lobo et al., 2021
Butyric acid^c^ (butanoic acid/ethyl acetic acid)	0.002^d^ 0.0–0.027^e^	0.173^g^, ^j^	Cheesy Butter‐like, cheese, floral	Rosend, Kuldjärv, Rosenvald, et al., 2019 Simonato et al., 2021
Isovaleric acid^c^ (3‐methylbutanoic acid/ β‐methylbutyric acid)	3.5–4.7^k^ 0.069–0.235	3^h^ 0.033^j^	Fatty, rancid	Simonato et al., 2021
Hexanoic acid^c^ (hexanoic acid/caproic acid)	0.047–0.119^d^ 0.008–0.011^f^	0.42^g^, ^j^ 3^h^	Sour, cheesy Cheese, fatty Fatty, cheese, rancid	Kliks et al., 2021 Ferreira et al., 2000 Simonato et al., 2021
Octanoic acid^c^ (octanoic acid/caprylic acid)	0.238–0.508^d^ 0.598–0.873^f^	0.5^g^, ^j^	Oily, waxy Cheese, fatty acid, rancid, harsh Fatty, cheese, rancid	Kliks et al., 2021 Li et al., 2020b Simonato et al., 2021
Decanoic acid^c^ (decanoic acid/capric acid)	1.1–3.8^k^	1^g^, ^j^ 15^h^	Rotting citrus	Kliks et al., 2021

*Note*: mg/L = ppm—parts per million.

Abbreviation: IUPAC, International Union of Pure and Applied Chemistry.

^a^ Identified using pure standards (at a concentration of 2.94 μ/L).

^b^ Within ethanol/water matrix as pH 3.2.

^c^ Compounds are also classified as fatty acids.

^d^ Medina et al. (2020).

^e^ Rosend, Kuldjärv, Rosenvald, et al. (2019)—using gas chromatography coupled to time‐of‐flight mass spectrometry (GC–TOF‐MS).

^f^ Li et al. (2020b)—using headspace solid‐phase microextraction coupled to gas chromatography with flame‐ionization detection (HS‐SPME–GC–MS/FID).

^g^ Ferreira et al. (2000)—using triangular tests (*n* = 26)—known concentrations of odorants in synthetic wines with 11% (v/v) ethanol, 7 g/L glycerin, and 5 g/L tartaric acid, pH adjusted to 3.4 with 1 M NaOH.

^h^ Guth (1997)—using triangular tests (*n* = 6)—known concentration of odorants in water/ethanol (90:10, v/v).

^i^ San Juan et al. (2012)—aroma threshold calculated in the laboratory: orthonasal thresholds were calculated in a 10% water/ethanol mixture containing 5 g/L of tartaric acid at pH 3.2.

^j^ Herrero et al. (2016)—aroma threshold values used are described by San Juan et al. (2012).

^k^ Sánchez et al. (2014)—concentration of volatile compounds identified in commercial ciders.

^l^ Simonato et al. (2021)—using gas chromatography–mass spectrometry (GC–MS).

^m^ Valles et al. (2005)—using gas chromatography–flame ionization (GC–FID).

Cider aroma and flavor, including RFAs, are shaped by the complex interactions of volatile and phenolic compounds, as well as the balance of sugars and acids (Jönsson & Nybom, 2006; Kim et al., 2023). Many RFAs were bred for fresh consumption rather than cider production, often misaligning with sensory expectations of cider apples (Espley et al., 2013). Recently, flavorful RFAs such as *Weirouge* (Germany, Italy), *Baya Marisa* (Germany), *Redlove* (Switzerland), and *Rosette* (England) have emerged as promising cider options (Bars‐Cortina et al., 2017). Further breeding is needed to enhance key sensory attributes for optimal RFA cider production (Yuste et al., 2022).

### 3.4 Texture and mouthfeel

Apple flavor and texture evolve at different rates based on variety and storage (Dixon & Hewett, 2000). Texture and mouthfeel, defined by multiple descriptors, are multiparameter sensory properties (Szczesniak, 2002). In this review, texture and mouthfeel will be used to describe the tactile properties of solid (i.e., apples) and liquid (i.e., apple juice and ciders) products, respectively.

RFAs exhibit distinct textural characteristics compared to WFA commercial varieties. Contessa and Botta (2016) found RFAs had higher flesh firmness (*p* < .05; 7.80 kg/cm^2^) than commercial varieties such as *Galaxy Gala* (6.93 kg/cm^2^), *Golden Delicious* (6.68 kg/cm^2^), and *Redchief* (6.40 kg/cm^2^). However, Espley et al. (2013) reported similar firmness between RFAs and *Royal Gala* apples when measured by a penetrometer (7.8 vs. 7.9 kg‐force). This variability may stem from anthocyanin, which can accelerate ripening and soften texture by reducing cell wall stiffness through pectin solubilization (Călugăr et al., 2021; Juhnevica‐Radenkova et al., 2014). While softer texture may be a drawback for RFAs in fresh consumption, fruit texture plays a pivotal role in juice and cider production too, as firmer apples yield more juice and affect aromatic compound extraction, influencing the final sensory profile (Chitarrini et al., 2021; Hampson et al., 2000). Therefore, apple texture remains critical for processing.

Beyond texture, mouthfeel plays a vital role in how beverages are perceived and is essential for the acceptance of consumers (Guinard & Mazzucchelli, 1996). Cider mouthfeel perception is due to interactions between volatile compounds and their effect on sourness, sweetness, bitterness, and astringency (Symoneaux et al., 2015). However, bitterness and astringency are the dominant drivers of mouthfeel in cider, largely dictated by phenolic composition (Alonso‐Salces et al., 2001, 2004; Guyot et al., 1998) (Table 1). Due to the inherently high concentration of phenolic compounds in RFAs (Section 2.3), it is widely accepted that most of these varieties would have a higher astringency compared to commercial dessert apples (Valois et al., 2015). The heightened astringency in RFAs can be attributed to an elevated concentration of procyanidins rather than the presence of anthocyanins, as the latter compounds generally contribute little to no taste or astringency (Brossaud et al., 2001; Espley et al., 2013).

While RFAs may not stand out for their eating quality, their distinct physicochemical and sensory properties show promise for cider production. A comprehensive investigation of how these properties change during cider processing is imperative to optimizing cider quality and expanding the market.

## 4 CHANGES IN PHYSICOCHEMICAL AND SENSORY PROPERTIES OF APPLES DURING CIDER PROCESSING

Ciders are composed of a complex mixture of nonvolatile (sugars, organic acids, and phenolic compounds) and volatile compounds that largely define their sensory properties (i.e., appearance, taste, aroma, flavor, and texture/mouthfeel). The cider‐making process typically involves three to four main stages: apple crushing, pressing, and sometimes concentrating the juice to approximately 70°Brix (reconstituted for fermentation), followed by the most important step, namely fermentation (Cousin et al., 2017; Nešpor et al., 2019). The latter encompasses alcoholic fermentation, in which sugars are converted to ethanol by yeast (e.g., *S. cerevisiae* and *S. bayanus*), and MLF, which can occur during maturation and is facilitated by lactic acid bacteria (LAB; e.g., *Oenococcus oeni*, *Lactobacillus* spp., and *Pediococcus* spp.) (Cousin et al., 2017; Madrera et al., 2010; Merwin et al., 2008) (Section 4.7.1). MLF is not typically desired in traditional cider production. While MLF is commonly used for deacidification (conversion of malic acid to lactic acid) (Swiegers et al., 2005), cider makers typically aim to maintain a certain level of acidity for the overall crispness and balance in ciders. This is particularly important for RFAs, known for their higher acid concentrations (Section 2.2). Exceptions exist, as some modern cider makers may intentionally introduce MLF to achieve specific flavor profiles, depending on their stylistic choices (Călugăr et al., 2021; Laaksonen et al., 2017; Peng et al., 2021; Sánchez et al., 2014).

**FIGURE 2:**
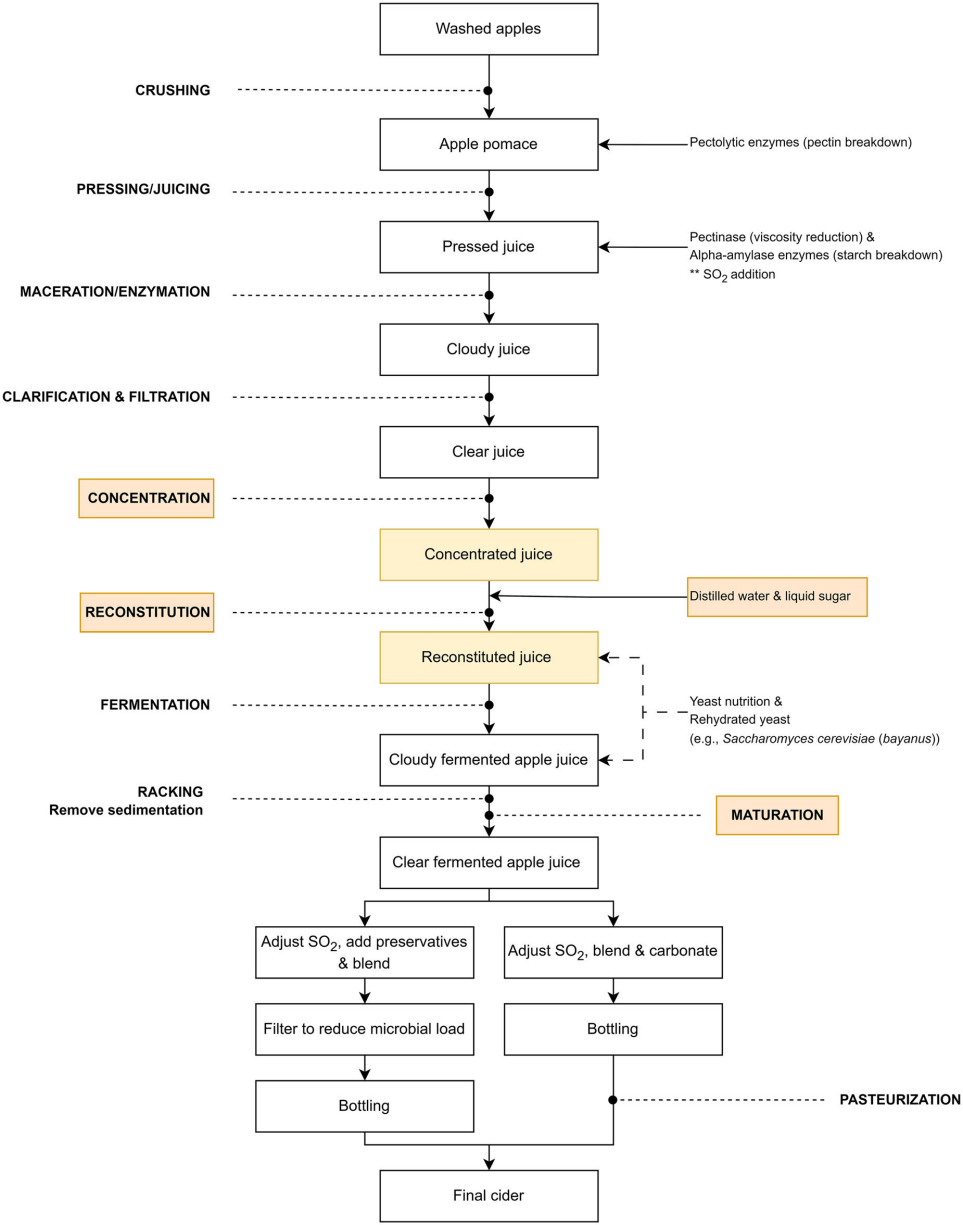
Typical flow diagram for modern‐day cider production, illustrating the transformation of apples into the final cider. The highlighted steps (yellow/orange) of juice concentration, reconstitution, and maturation are optional techniques but are not integral to all cider production methods. Many postfermentation paths exist (after clear fermented apple juice), including SO_2_ adjustment, blending, bottling, and pasteurization, leading to different cider styles.

A visual representation of the modern‐day cider‐making process, detailing key stages, along with various iterations of the process, has been included in Figure 2. While adhering to the fundamental cider‐making steps is essential for crafting a high‐quality cider, the creation of a unique product relies on nuanced adjustments and optimal execution of the different cider‐making steps. Therefore, producers can consistently craft high‐quality ciders by understanding the physicochemical and sensory changes occurring during the complex stages of cider production.

### 4.1 Apple pre‐ and postharvest effects

The maturity of apples at harvest greatly affects the apple quality, storage performance, and final cider quality (Wang & Cheng, 2011). In a meta‐analysis of apple fruit and juice quality traits for potential use in cider production, 80%–100% starch degradation was determined to be the optimal maturity for cider production (VanderWeide et al., 2022). Apples that are harvested too early could have a sour and starchy taste, while apples that are harvested too late may be soft and mealy (Riekstina‐Dolge et al., 2012). Although some of these characteristics are irrelevant if apples are used for ciders, high starch content in apples (>50% starch by DW) may lead to juice clarification issues, which producers naturally want to avoid (Riekstina‐Dolge et al., 2012; Travers et al., 2002).

Knowledge of the distribution and evolution of phenolic compounds during the ripening of RFAs can be helpful for consumers and the food industry to maintain elevated levels of bioactive compounds in their ciders. A study by Alberti et al. (2017) aimed to investigate the effects of variety and ripening stages on the distribution of phenolic compounds and antioxidant capacity in dessert apples (*Fuji Suprema*, *Gala*, and *Eva*). Studies on the changes in phenolic composition during apple development report that large amounts of phloridzin, catechin, and hydroxycinnamic acid derivatives (e.g., chlorogenic acid) are formed early in fruit development (Spanos & Wrolstad, 1992). However, during rapid fruit growth, the concentrations of these compounds decrease dramatically, and this decline continues as the fruit ripens (Awad et al., 2001). While some phenolic compounds decrease in concentration, the anthocyanin idaein (responsible for the red color in RFAs) accumulates during maturation (Awad et al., 2001). Subsequently, ripening may lead to variation in the concentration of different phenolic compounds (Section 2.3).

Previous research has shown that apple ripeness affects phenolic content and volatile composition. Ciders made from overripe apples contain more volatile compounds, such as higher alcohols (e.g., propanol, isobutanol, butanol, isoamyl alcohol, hexanol, and 2‐phenyl ethanol) and acetate esters (e.g., ethyl acetate, isoamyl acetate, ethyl butyrate, and ethyl hexanoate), compared to those made from less ripe apples (Rosend, Kuldjärv, Rosenvald, et al., 2019) (Section 4.7.2). Additionally, a study comparing the sensory properties of ciders made from machine‐ or hand‐harvested *Brown Snout* apples highlighted the importance of harvesting fully matured fruit to ensure uniform raw material for cider production (Alexander et al., 2018).

The importance of apple ripeness in cider production cannot be overstated, as it greatly influences the volatile and phenolic profiles of the finished product. Notably, the incorporation of RFAs offers an intriguing dimension, offering the potential to enhance the physicochemical and sensory properties of ciders.

### 4.2 Crushing

Crushing (Figure 2) refers to physically breaking the apples into smaller pieces and rupturing the cells for easier release of juice during pressing. Since the crushing process results in the surface area of the material being exposed to air, immediate oxidation catalyzed by PPO occurs, leading to browning (Charles‐Rodríguez et al., 2007; Nicolas et al., 1994). The browning of fruits and fruit juices is associated with the oxidation of phenolic compounds (Nicolas et al., 1994; Spanos & Wrolstad, 1992). RFAs, such as the variety *Weirouge*, whose vivid red color is imparted by anthocyanins, are a particularly challenging fruit regarding oxidation damage during processing (Wagner et al., 2022).

During crushing, some producers apply high temperatures (40–70°C) to obtain a higher juice yield (especially in firmer varieties) and reduce processing time, but these treatments are always accompanied by increased energy consumption and loss of juice quality (e.g., decreased vitamin content, color, and flavor changes) (Charles‐Rodríguez et al., 2007; Genovese et al., 1997; Gerard & Roberts, 2004; Grimi et al., 2011). Regardless of the crushing method, it remains destructive, and many volatiles and phenolic compounds are either lost (due to poor extractability or evaporation at increased temperatures) or negatively influenced (due to oxidation) during this process (Alberti et al., 2016; Arvisenet et al., 2006; Călugăr et al., 2021). To avoid these consequences but still have high juice yields, other nonconventional and nonthermal treatments such as microwave, pulse electric fields, enzymatic treatments, ultraviolet treatments, and ultrasound have been proposed (Călugăr et al., 2021; Charles‐Rodríguez et al., 2007). These nonconventional treatments used alone or in combination with conventional crushing methods offer promising avenues for improving RFA juice yields while preserving the phenolic and volatile compounds during production.

### 4.3 Maceration/enzymation

Maceration is the technological process of allowing the apple pomace after crushing to oxidize before pressing (Călugăr et al., 2021; Nogueira et al., 2008). Maceration is a technique used by the juice industry, in conjunction with enzymatic treatments (Figure 2), to increase total juice output and simplify the pressing process (Malec et al., 2014; Mihalev et al., 2004). During maceration, juice color develops due to oxidation, and pectin leaches out of the cell walls, after which naturally occurring enzymes break it down to increase the juice yield, leaches out of the cell walls (Alonso‐Salces et al., 2004; Lea & Piggot, 1996). Previous research has shown great variation in times and temperatures used for maceration including 1 h at 2°C (Villière et al., 2015), 1 h at 20 ± 3°C (Mihalev et al., 2004), 2–4 h at 15°C (Way et al., 2019), 24 h at 20°C (Vidrih & Hribar, 1999), and 7 days at 25°C (Budak et al., 2015). Consequently, there are contradictory results regarding the effect of maceration on the phenolic and volatile content of cider. A study by Vidrih and Hribar (1999) found that maceration (24 h at 20°C) increased the synthesis of volatile compounds such as butanol, isobutanol, propanol, methanol, and phenethyl alcohol. However, these higher alcohols may also negatively influence the aroma and taste of the final cider (Section 4.7.2). Furthermore, the oxidation process during maceration (1 h at 20 ± 3°C) may cause a higher proportion of procyanidins (tannins) to be adsorbed by the apple pomace's cell‐wall matrix (close to 32%), hindering their extraction during pressing and resulting in a reduction in the phenolic content of the juice and subsequent cider (Mihalev et al., 2004; Nogueira et al., 2008). The most recent study by Way et al. (2019) found that maceration (2–4 h at 15°C) reduced the phenolic extraction of apple varieties with low phenolic content (e.g., dessert varieties *Pink Lady*, *Red Delicious*, and *Sturmer*: 0.145–0.786 mg/L measured with Folin–Ciocalteu assay), while varieties with high phenolic concentrations (e.g., cider variety *Bulmer's Norman*: 1.257–3.105 mg/L) showed increased extraction.

The disparities in maceration time and temperatures across studies contribute to different conclusions by various authors. However, regardless of these variations, it is evident that an extended period of oxidation between crushing and pressing results in an overall decrease in phenolic compounds likely caused by PPO‐catalyzed and other oxidation reactions (Mihalev et al., 2004; Nogueira et al., 2008; Van der Sluis et al., 2001). Naturally, avoiding prolonged oxidation/maceration is essential, since it may lead to deterioration in juice quality and subsequently impact the cider quality as well (Călugăr et al., 2021; Cliffe et al., 1994; Singleton et al., 1974; Way et al., 2019).

Enzymatic treatment of apple pomace is a common practice in the juice and concentration industries, typically applied after crushing and/or maceration (sometimes during maceration) to reduce mash viscosity and water‐binding capacity caused by naturally occurring pectin (Kumar, 2015; Will et al., 2002) (Figure 2). This facilitates easier juice extraction during pressing and enhances the efficiency of presses or decanters (Grassin & Coutel, 2010). The enzyme application conditions (i.e., time and temperature) are largely dependent on the specific enzymes used, with current trends favoring reduced temperature and time to lower processing costs (Markowski et al., 2009). Pectinases (enzymes that hydrolyze pectins) are typically added to crushed apples before pressing or juice extraction to help separate flocculent precipitates through sedimentation (i.e., clarification process), filtration, or centrifugation (Kumar, 2015; Ribeiro et al., 2010). Then, the apple pomace is liquefied with a mixture of pectinases and cellulases (enzyme complex used to degrade cellulose) for complete juice extraction (Călugăr et al., 2021; Will et al., 2000). Besides promoting juice extraction, various researchers have shown that the addition of pectolytic enzymes also increases the release of various phenolic compounds (up to twice as much) and other nutritionally important components from the apple flesh particles, which may improve the sensory properties of the final cider (Kumar, 2015; Markowski et al., 2009; Mihalev et al., 2004; Will et al., 2002). For RFAs, enzymatic treatment is important as it enhances phenolic richness and imparts unique flavor characteristics to the cider.

### 4.4 Pressing/juicing

Following enzymation, the apple juice is mechanically expressed by pressing or decanting (i.e., centrifugation) (Călugăr et al., 2021; Grimi et al., 2011; Will et al., 2002) (Figure 2). The amount of pressure used to press the crushed fruits determines the pressing fraction, which in turn affects how much phenolic compound extraction occurs. The bark and seeds may be crushed under greater pressure, releasing tannins (Călugăr et al., 2021; Way et al., 2019). Similar to crushing and maceration, enzymatically catalyzed polyphenol oxidation (20%–60%) occurs during pressing (Călugăr et al., 2021; Guyot et al., 2003; Nicolas et al., 1994; Zardo et al., 2013). Since up to 90% of an apple's tannins are lost between milling and pressing, small changes in processing can dramatically improve tannin extraction and retention in RFA cider, subsequently enhancing the final cider quality (Van der Sluis et al., 2001).

The pressing technique has been shown to affect the concentration of volatile compounds in apple juice. For example, if pressing is done at a lower speed and a lower temperature (2 vs. 15°C), the acetates (butyl acetate, hexyl acetate, and phenethyl acetate) may be present in higher proportions (*p* < .05) in the apple juice (Călugăr et al., 2021; Villière et al., 2015). At the same time, the amount of pressure applied and the duration of skin contact influence the extraction of aroma compounds and precursors. Effective management of pressing conditions is crucial in RFA cider production, as it influences the extraction of phenolic compounds that define sensory properties. Optimizing these conditions can enhance phenolic richness and unique aromas, advancing modern cider production.

### 4.5 Clarification and filtration

The turbidity (i.e., cloudiness) of apple juice or the finished cider is the result of smaller materials such as precipitated polyphenols, carbohydrates (pectins), and proteins or larger particles such as yeast and bacterial cells suspended in the product (Choi & Nielsen, 2005; Ma et al., 2018; Peng et al., 2021). Clear and cloudy apple juice can be used to make cider, but often the juice is at least roughly filtered before fermentation (Figure 2). If desired and required, the finished cider can be subjected to further clarification treatments (i.e., racking) (Lea & Piggot, 1996). The clarification of apple juice or cider is done to improve the appearance and stability of the product (Al Daccache et al., 2020; Alexandre & Charpentier, 1998; Moulton & Zimmerman, 2010). Common clarification techniques include fining (sedimentation and settling), centrifugation, and clarification with separators or filters (Alexandre & Charpentier, 1998; Marks et al., 2007b).

A disadvantage of clarification is that various polyphenols, sometimes desirable for aroma, flavor, and health benefits, are also removed (Lea & Piggot, 1996; Oszmiański et al., 2007). A study by Markowski et al. (2009) found that the total phenolic content in dessert apple juice after depectinization and filtration with diatomaceous earth decreased by about 10% in two juices. Another study found that all of the clarification techniques (static settling, pectinase treatment, and centrifugation) tested on cloudy apple juice decreased (*p* < .05) the concentration of total phenolic compounds from 60% to 30% (Ma et al., 2018). The clarified juice was fermented to cider, and the same clarification techniques were employed. While the total phenolic compounds in the cider made from the clarified juice were much lower (240 vs. 630 mg GAE/L), the three clarification techniques had no effect (*p* > .05) on the total phenolic content of the cider (Ma et al., 2018). This finding suggests that the primary phenolic loss occurs during the initial juice clarification step rather than filtration after fermentation. The phenolic compounds are structurally integrated within the apple matrix and may persist despite clarification efforts (Rocchetti et al., 2022). Furthermore, enzymatic clarification with pectinase has also been shown to reduce yeast assimilable nitrogen without affecting polyphenol composition in cider, indicating selective retention of soluble phenolics rather than their complete removal (Ma et al., 2018). The stability of polyphenols post‐clarification may also be influenced by pH and solubility dynamics (Sanoner et al., 1999), further explaining why the overall polyphenol composition in cider remains unaffected.

Alonso‐Salces et al. (2004) found that employing centrifugation (10,000 rpm, 15 min at 4°C) on the crude apple juices during the cider‐making process with *Basque* cider apple varieties resulted in a decreased procyanidin content. This reduction was attributed to the capacity of procyanidins to precipitate proteins and interact with the polysaccharides of the cell wall. The interaction of tannins with the cell wall polysaccharides also inhibits the pectolytic enzymes involved in the clarification process (Guyot et al., 2003).

In navigating the complex domain of cider production, the optimization of clarification techniques both before and after fermentation is important in shaping ciders characterized by increased phenolic content (as with RFAs) and balanced volatile profiles. This would contribute to the continuous improvement and enhancement of the cider industry, specifically considering the move toward the production of ciders made from novel RFAs.

### 4.6 Concentration

Concentrating apple juice for cider production reduces storage, packaging, and transportation costs (Bozkir & Baysal, 2017). Furthermore, the technique produces juice that is more chemically and microbiologically stable (Bozkir & Baysal, 2017). The use of apple juice concentrate for cider production also results in more standardized fermentation kinetics and cider quality compared to using fresh apple juice. However, fermentation using reconstituted apple juice concentrate may require additional nutrient supplementation to compensate for the loss of some nutrients during concentration and to support the viability of the yeast cells (Rosend et al., 2020).

Unconcentrated fermented apple juice has been reported to exhibit higher levels of natural antioxidants, including polyphenols, as well as superior aroma and flavor compared to apple juice derived from concentrate (Braddock & Goodrich, 2002; Park et al., 2020). This is likely due to traditional concentration techniques (e.g., thermal evaporation, vacuum evaporation, rotary evaporation, and freeze concentration) that lead to considerable losses of phenolic compounds, vitamins, and volatile compounds (Bozkir & Baysal, 2017; Guyot et al., 2003). However, direct quantitative comparisons of phenolic content and antioxidant activity before and after concentration remain limited, hindering verification of these reported effects.

The concentration of apple juice may also lead to the formation of a cooked taste and potential carcinogenic compounds such as furans or hydroxymethyl furfural (HMF) (Bozkir & Baysal, 2017). Another disadvantage is the fact that volatile compounds can be lost during concentration techniques such as vacuum evaporation, leading to a reduction in the sensory quality of products such as fruit juices. In the case of fruit juices, some of the original juice can be added to the concentrate during the process known as “cut‐back juice” to compensate for losses. Alternatively, the volatile compounds can be separated from the vapor, concentrated, and added to the juice concentrate. Furthermore, membrane filtration technologies (such as ultrafiltration) and membrane concentration techniques (including osmotic distillation [OD] and membrane distillation [MD]) offer advantages over traditional concentration methods. A study by Onsekizoglu et al. (2010) found that in comparison with thermal evaporation, OD and MD preserved the natural color and aroma (particularly *trans*‐2‐hexenal concentration) of apple juice while maintaining its phenolic content, with no development of off‐flavors (i.e., 5‐HMF).

Concentrating apple juice for cider production introduces complexities that impact both the chemical and sensory properties of the final product. As the cider industry navigates these considerations, a balanced and informed approach to concentration techniques becomes imperative to ensure the preservation of the unique properties that RFAs have to offer in cider production.

### 4.7 Fermentation

The single most crucial step in the production of cider is alcoholic fermentation (Figure 2). The fermentation process is initiated in the presence of oxygen and begins a few hours after inoculation with the yeast (e.g., *S. cerevisiae*, *S. bayanus*, *S. uvarum*, and a variety of non‐*Saccharomyces* yeasts) (Heikefelt, 2011). This process triggers a series of physicochemical transformations (Section 4.7.1) that depend on the composition of the apple juice, microbial activity, and processing conditions (Călugăr et al., 2021; Riekstina‐Dolge et al., 2012). The raw material plays the most significant role in fermentation, which relates to the sensory quality of the final product (Al Daccache et al., 2020; Beltran et al., 2008; Cousin et al., 2017; Laaksonen et al., 2017; Rosend, Kuldjärv, Rosenvald, et al., 2019; Way et al., 2022). The complex changes of the apple juice during fermentation can further be convoluted by altering fermentation conditions such as temperature, sugar concentrations, available nutrients, oxygen exposure, pH levels, the fermentation vessels, fermentation duration, inoculation method (i.e., spontaneous/wild fermentations or controlled yeast inoculation), and yeast strains (Beltran et al., 2008; Călugăr et al., 2021). Changes in any of these parameters will affect the final nonvolatile and volatile compounds, which subsequently influence the sensory properties of the cider.

A thorough understanding of metabolic pathways (Section 4.7.1) is crucial for regulating fermentation efficiency and shaping the cider's sensory profile. Additionally, a well‐defined apple composition and careful yeast selection (Section 4.7.3) help navigate fermentation complexities, ensuring optimal yeast performance, balanced aroma development (Section 4.7.2), and the prevention of off‐flavors (Rosend, Kuldjärv, Arju, et al., 2019).

#### 4.7.1 Yeast metabolism

Cider fermentation involves a sequence of metabolic processes that influence the final composition and sensory properties of the beverage (Beltran et al., 2008). The interaction between the varietal characteristics of the fruit and the yeast's ability to produce these compounds contributes to the sensory complexity of fermented beverages such as ciders, in contrast to the simpler, sweeter fruit juices from which they are derived (Swiegers et al., 2005). Yeast metabolism encompasses the biochemical processes of assimilation (energy‐consuming) and dissimilation (energy‐generating) of nutrients within yeast cells (Walker, 1998). The process starts with sugar metabolism (glycolysis), where glucose and fructose are broken down into pyruvate via key enzymes such as hexokinase, phosphofructokinase, and enolase (Bisson, 1999). This step generates ATP (adenosine triphosphate) and NADH (nicotinamide adenine dinucleotide phosphate), which provide energy for yeast growth (Walker & Stewart, 2016).

Alcoholic fermentation follows, during which pyruvate is decarboxylated by pyruvate decarboxylase to produce acetaldehyde, which is then reduced to ethanol by alcohol dehydrogenase (Guittin et al., 2023; Kavvadias et al., 1999). This reaction also releases CO₂, which contributes to the carbonation of the cider, providing its characteristic effervescence (Wilson et al., 2021). After alcoholic fermentation is completed, LAB performs MLF, which is related to an increase in pH and changes in the aromatic and mouthfeel properties (Herrero et al., 1999; Sánchez et al., 2014; Sumby et al., 2010). Li et al. (2021) compared the effects of simultaneous (SIM) and sequential (SEQ) MLF on RFA cider. They found that both MLF methods increased the pH by approximately 0.25 units and significantly decreased (*p* < .05) the malic acid concentration (550 mg/L in SEQ and 650 mg/L in SIM) compared to alcoholic fermentation alone (5810 mg/L). Given that RFAs are generally more acidic due to their higher malic acid content, MLF could play a crucial role in modulating their sensory properties (Section 2.2). Quantitative descriptive analysis revealed that ciders produced by SIM and SEQ had improved sensory quality (specific values for attributes not provided), with the SIM ciders scoring the highest for floral and fruity notes (Li et al., 2021). While these findings suggest the potential benefits of SIM for enhancing the sensory attributes of RFA cider, further research is needed to confirm its effectiveness across different apple varieties, fermentation conditions, and microbial compositions.

The tricarboxylic acid cycle, which follows glycolysis, plays a central role in yeast metabolism by oxidizing acetyl‐CoA (co‐enzyme A) to generate ATP, NADH, and key metabolic intermediates (Wang et al., 2022). These intermediates influence yeast growth, fermentation efficiency, and the production of organic acids and aroma compounds (Dixon & Hewett, 2000). Additionally, citric acid metabolism may result in diacetyl production, contributing a buttery aroma that can affect the overall sensory profile of cider (Sumby et al., 2010).

Phenolic transformations begin during alcoholic fermentation and continue throughout the fermentation process. These transformations, which involve enzymatic oxidation, condensation, and polymerization, affect cider's astringency, mouthfeel, and color stability (Leonard et al., 2021). For example, anthocyanin pigments may degrade or form stable complexes during fermentation, impacting the final appearance of the cider (Cai et al., 2022). The retention of these pigments is of particular interest in RFA ciders, as color stability contributes to consumer appeal (Section 3.1). Finally, volatile compound formation during fermentation greatly impacts the aroma and flavor of cider (Section 3.3). The formation of these compounds, influenced by yeast metabolism and interactions with phenolic compounds, will be discussed in more detail in Section 4.7.2.

While cider fermentation from traditional apple varieties is well understood, the impact of RFAs on yeast metabolism remains underexplored. No studies have directly compared the fermentation dynamics of RFAs with those of WFA varieties, making it difficult to predict how these differences in fruit composition may affect fermentation and cider quality. Given that yeast metabolism can vary depending on the strain, this review provides a general overview of the process, with strain‐specific variations left for further research. For a more detailed exploration of *S. cerevisiae* metabolic pathways in beverage fermentations, the reader is referred to Hirst and Richter (2016).

#### 4.7.2 Volatile compounds formed during fermentation

Volatile compounds, influenced by apple variety and yeast metabolism (Section 4.7.1), play a key role in shaping the aromatic profile and sensory quality of ciders and other fermented beverages (Bingman et al., 2020; Kliks et al., 2020). New compounds such as alcohols, esters, aldehydes, and ketones are formed during fermentation, suggesting an improvement in aroma complexity in the fermented product (Chen et al., 2019; Wu et al., 2020; Ye et al., 2014). A study using response surface methodology (RSM) identified optimal fermentation parameters for *Huaniu* (derived from *Red Delicious* varieties) apple cider, which could serve as a reference for broader cider production (Mu et al., 2023). The optimal conditions included a fermentation temperature of 25.48°C (using *S. cerevisiae* 1023), initial soluble solids of 18.90°Brix, an inoculation amount of 8.23%, and an initial pH of 3.93. Under the optimized conditions, a total of 72 distinct volatile compounds were identified, including 41 esters, 16 alcohols, six acids, and nine other substances. Notably, esters were found in abundance, with ethyl acetate (4.198 mg/L), ethyl octanoate (0.991 mg/L), ethyl caproate (0.132 mg/L), and isoamyl acetate (0.115 mg/L) being particularly prevalent. Alcohols were also present in great amounts, including 3‐methyl‐1‐butanol (1.157 mg/L), 2‐phenyl ethanol (0.391 mg/L), 2‐methyl‐1‐propanol (0.234 mg/L), and hexanol (0.209 mg/L). Additionally, key acids, acetic acid (0.516 mg/L), caproic acid (0.124 mg/L), and octanoic acid (0.053 mg/L), contributed to the overall aromatic profile. The impact of these compounds on the sensory properties of ciders, including their positive or negative effects at certain concentrations, will be explored in the subsequent sections of this review.

Dixon and Hewett (2000) composed a comprehensive table summarizing important apple volatile compounds and their sensory descriptors and reviewed their aroma threshold values. However, these comparisons have not been made for fermented apple products such as ciders. Table 2 summarizes the relative concentration of volatile compounds previously identified in ciders from any apple category/variety mostly using headspace solid‐phase microextraction coupled with gas chromatography with flame‐ionization detection (HS‐SPME/GC–FID). Table 2 includes the common names with International Union of Pure and Applied Chemistry (IUPAC) nomenclature and alternative names, concentrations in ciders, perception thresholds, and aroma descriptions for each volatile compound.

Although some apple varieties have been distinguished from one another based on their chemical composition, there is no conclusive information on the effects of different apple categories on the volatile composition of cider (El Hadi et al., 2013; Rosend, Kuldjärv, Rosenvald, et al., 2019). While ciders are characterized by hundreds of aromatic constituents present in small amounts in the apples, the characteristic volatiles differ among dessert, cider, and RFA varieties (Hinkley et al., 2021; Merwin et al., 2008). Several studies identified and quantified the volatile compounds in ciders made from dessert apples (Dos Santos et al., 2018; Wilson et al., 2021; Ye et al., 2014), but much fewer studies focused on ciders from cider apples (Perestrelo et al., 2019). Furthermore, research on the volatile profile of RFAs is limited, and no studies have investigated the volatile compounds of cider made from RFAs. In fact, Li et al. (2020b) were one of the first to report on the dynamic evolution of volatile compounds during the production of alcoholic beverages (through co‐fermentation and MLF) from RFAs.

Understanding the formation and concentration of volatile compounds during fermentation is essential for cider makers. This knowledge allows producers to identify key compounds that shape the sensory properties and final cider quality. By knowing which compounds form during RFA fermentation, cider makers can consistently craft ciders with distinctive qualities, driving innovation and offering tailored sensory experiences to consumers.

##### Alcohols

In ciders and other fermented alcoholic beverages, ethanol is the primary volatile compound resulting from alcoholic fermentation (Section 4.7.1), followed by higher alcohols (mainly ethyl acetate, isoamyl alcohol, and 2‐phenyl ethanol) (Chen et al., 2019; He et al., 2022; Herrero et al., 2016; Mangas et al., 1996; Nešpor et al., 2019; Satora et al., 2008; Ye et al., 2014) (Table 2). Alcohols are directly derived from yeast metabolism primarily by the conversion of the branched amino acids (leucine, isoleucine, and valine) through the Ehrlich pathway (Cousin et al., 2017; Dos Santos et al., 2016; Hirst & Richter, 2016; Rous & Snow, 1983; Swiegers et al., 2005; Vidrih & Hribar, 1999) (Section 4.7.1). Subsequently, the availability of specific amino acids during fermentation plays a crucial role in determining the concentration of these higher alcohols in the final product (Mangas et al., 1994).

In ciders, higher alcohols are mostly represented by 2‐phenyl ethanol and iso‐pentanols, followed by isobutanol, propanol, butanol, isoamyl alcohol, and hexanol (Lea & Piggot, 1996; Nešpor et al., 2019; Wang et al., 2020; Zhao et al., 2014) (Table 2). The concentration of higher alcohols in ciders is influenced by the apple variety, the juice extraction method, fermentation parameters (including the type of yeast strain—e.g., *Saccharomyces* spp., non‐*Saccharomyces* spp., and LAB), and the clarification technique (Călugăr et al., 2021; Magalhães et al., 2017; Perestrelo et al., 2019; Romano & Suzzi, 1993). For example, the presence of insoluble particles in apple juice (e.g., cloudy/turbid juice) increases the content of higher alcohols (i.e., isoamyl, 2‐phenyl ethanol, isobutanol, propanol, and butanol) in the cider (Beech & Carr, 1977; Vidrih & Hribar, 1999).

Higher alcohols may greatly affect the sensory properties of ciders, even if they constitute a relatively small amount of the total substances (Cousin et al., 2017; Vidrih & Hribar, 1999). Positive contributors to cider aroma and flavor that cider makers target include butanol, which enhances the aroma quality by improving the intensity, balance, and complexity of volatile compounds, thereby increasing overall consumer acceptance of apple juice (Cousin et al., 2017; Jepsen, 1978). Additionally, butanol and hexanol are associated with warm or sweet characteristics, propanol and isobutanol give cider an alcoholic flavor, and 2‐phenyl ethanol is an important contributor to the typical cider flavor while also imparting floral aromas (Beech, 1972; Červenčík et al., 2022; Guiné et al., 2021; Lambrechts & Pretorius, 2000) (Table 2). In general, at concentrations above 400 mg/L, some alcohols (e.g., isoamyl alcohol and 2‐phenyl ethanol) have been reported to develop harsh and undesirable flavors, which lead to consumer dissatisfaction (Rous & Snow, 1983; Vidrih & Hribar, 1999).

##### Acids and fatty acids

Acids are a class of volatile compounds that contribute to the complexity and fruity, aromatic balance of fermented apple juice and cider (Guiné et al., 2021). Volatile acids in alcoholic beverages are primarily formed during fermentation, with acetic acid being the most prevalent (75%–85%) (Satora et al., 2008). While ethanol is produced by yeast, acetic acid is primarily formed by acetic acid bacteria (*Acetobacter*) through the oxidation of ethanol, especially in the presence of oxygen (De Klerk et al., 2018). This is particularly evident in ciders with higher alcohol content, where acetic acid concentrations can increase due to bacterial activity (Kliks et al., 2021). A combination of nitrogen limitation (causes yeast stress) and high sugar concentrations (promotes bacterial activity) in apple juice can promote acetic acid production during fermentation (Satora et al., 2008). The high acetic acid concentration (>1200 mg/L) in some ciders can be attributed to the fact that yeast produces it in the presence of oxygen, and continuous fermentation allows oxygen to be continuously added to the medium (nonfermented apple juice) (Kliks et al., 2021; Shekhawat et al., 2017). Therefore, findings from Shekhawat et al. (2017), who investigated the impact of oxygenation on the performance of three non‐*Saccharomyces yeasts* (*Torulaspora delbrueckii*, *Lachancea thermotolerans*, and *M. pulcherrima*) in co‐fermentation with *S. cerevisiae*, suggest that to produce cider with acceptable qualities (i.e., a balanced profile with appropriate levels of sweetness, acidity, and tannins based on the type of cider [Beer Judge Certification Program, 2015]), the oxygen level in the apple juice needs to be carefully managed (e.g., using closed systems to maintain dissolved oxygen at ≤1%) during processing.

As with higher alcohols, fatty acids and other acids play an important role in the final flavor profile of fermented beverages such as ciders. Acetic acid contributes to a pungent, sharp, and vinegary aroma (Guiné et al., 2021; Liu et al., 2016; Xu et al., 2007), while decanoic and octanoic acids contribute to sweaty, cheesy, soapy, green, and fatty notes (Medina et al., 2020; Perestrelo et al., 2019; Xu et al., 2007; Ye et al., 2014) (Table 2).

##### Esters

Esters are the most important volatile compounds in ciders and greatly contribute to cider quality (Kliks et al., 2020; Perestrelo et al., 2019; Ye et al., 2014). Esters are mainly formed by the esterification of fatty acids (e.g., hexanoic acid, octanoic acid, and decanoic acid) with higher alcohols (Section 4.7.2.1) or ethanol via the Ehrlich pathway during fermentation (Hirst & Richter, 2016; Nešpor et al., 2019; Saerens et al., 2010; Samoticha et al., 2019). The biosynthesis of esters is influenced by several factors, such as aeration of the must, fermentation temperature, fermentation technique, and even the ripeness of the fruit (De la Roza et al., 2003). Although a great number of esters are found in apples and apple juice (Ortiz et al., 2011), the majority are created during the fermentation process as secondary metabolites (Ye et al., 2014). Alcohols, fatty acids, CoA, and an ester‐synthesizing enzyme react to produce esters during fermentation (Lea & Piggot, 1996; Zhao et al., 2014) (Section 4.7.1). Twenty‐two esters were detected in ciders made from *Fuji* apples, with the main compounds including ethyl acetate, ethyl caprate, ethyl caprylate, ethyl caproate, and isoamyl acetate (Table 2) (Wang et al., 2020). Various other studies investigating the volatile profiles of different ciders have found similar results (Bingman et al., 2020; Kliks et al., 2020; Medina et al., 2020; Nešpor et al., 2019; Perestrelo et al., 2019; Wilson et al., 2021).

A small change in the ester concentration of ciders can have a substantial impact on their final sensory quality (Cousin et al., 2017; Sumby et al., 2010). Esters mainly contribute to the fruity and floral aromas of fermented beverages (Cousin et al., 2017; Nešpor et al., 2019; Verstrepen et al., 2003). Specifically, ethyl esters provide the sweet, pineapple, apple, fruity, and alcoholic aromas that are characteristic of cider (Bingman et al., 2020; Rosend, Kuldjärv, Rosenvald, et al., 2019; Saerens et al., 2010; Wang et al., 2020) (Table 2). However, unlike other higher molecular weight ethyl esters, an excessive amount of ethyl acetate imparts an unpleasant solvent‐like aroma (Červenčík et al., 2022; Cousin et al., 2017; Simonato et al., 2021). In ciders, large amounts of ethyl acetate enhance the intensity of the vinegar aroma and flavor, considered a negative for cider quality (Beech & Carr, 1977; Satora et al., 2008; Williams, 1974). The acetate esters contribute to the banana aroma, apple flavor, honey, and floral aroma in ciders (Červenčík et al., 2022; Li et al., 2020b; Rosend, Kuldjärv, Rosenvald, et al., 2019; Simonato et al., 2021) (Table 2).

The vast array of esters identified in ciders plays a crucial role in shaping the aromatic profile and overall sensory experience of the beverage. Focused research on esters in RFA ciders could provide deeper insights into optimizing production techniques, enabling cider makers to better harness the distinctive qualities of RFAs, and offering consumers a broader and more refined selection of cider options.

##### Aldehydes and ketones

Aldehydes, and to a lesser extent ketones, are also considered major volatile compounds in ciders. Three to six aldehydes and one to 10 ketones have previously been identified in a variety of apple juices, fermented apple juices, and apple ciders (Hinkley et al., 2021; Kliks et al., 2020; Qin et al., 2018; Wu et al., 2020; Ye et al., 2014). However, the concentration of several aldehydes, including benzaldehyde, is considerably lower during fermentation than in the corresponding unfermented juice (Liu et al., 2020). This reduction is primarily due to yeast metabolism, which converts aldehydes into alcohols (alcohol oxidation) or acids (acid decarboxylation), while oxidation and enzymatic degradation further contribute to their decline during fermentation (Section 4.7.1). However, aldehydes may appear after LAB fermentation (Wu et al., 2020). In apple juice fermented by *Lactobacillus casei*, *Lactobacillus rhamnosus*, *Lactobacillus plantarum*, or *Lactobacillus acidophilus*, acetaldehyde concentrations measured 0.015–0.040 mg/kg, whereas it was not detected in the unfermented juice (Chen et al., 2019). Studies on RFAs have revealed notable variations in aldehyde composition, with hexanal and (*E*)‐hex‐2‐enal being the most abundant (Chitarrini et al., 2021). These aldehydes, known for their green and fruity notes, exhibited substantial differences across RFA genotypes, with hexanal levels ranging from 0.00312 to 0.0215 mg/L and (*E*)‐hex‐2‐enal from 0.006 to 0.014 mg/L. Such variability underscores the genetic influence on volatile compound formation in RFAs, which may result in unique sensory attributes compared to WFA varieties.

Although aldehydes (e.g., decanal, octanal, 2‐nonanal, and (*Z*)‐2‐heptanal) are only present in low amounts in fermented clarified apple juice (Peng et al., 2021), researchers found that they still contribute to the aroma of the product due to very low olfactory thresholds (0.500 mg/L; Table 2) (Chitarrini et al., 2021; Guiné et al., 2021). Aldehydes, which typically contribute to a distinctive fruity and herbaceous aroma in juice and traditional ciders (Chen et al., 2019; Valappil et al., 2009), can have a negative impact on the aroma of fermented products at high concentrations (>200 mg/L) due to the activity of acetic acid bacteria (e.g., *Acetobacter aceti*) and/or oxidation processes (Gil et al., 2006; Qin et al., 2018) (Section 4.7.2.2). Acetaldehyde, in particular, could even possess a pungent and irritating aroma (Miyake & Shibamoto, 1993). Furthermore, during cider storage, the fresh green aroma can shift to more perfume‐like aromatic compounds, a change often associated with the presence of esters (Kühn & Thybo, 2001). Monitoring aldehyde levels is crucial for ensuring the quality of RFA ciders, especially as their persistence and interactions with other volatiles postfermentation remain underexplored.

Acetaldehyde can also act as a precursor for the major ketone in ciders, acetoin (formed by the oxidation of diacetyl), which explains why these two compounds are commonly associated with each other in fermented beverages (Han et al., 2019). However, the elevated concentrations of both compounds in ciders may be due to incomplete conversion, as acetoin can be produced by yeast (e.g., *S. cerevisiae*) and LAB (e.g., *Lactobacillus* spp.) during fermentation (Haider et al., 2014; Han & Du, 2023) (Section 4.7.1). The majority of ketones (e.g., 2‐octanone, 6‐methyl‐5‐hepten‐2‐one, and geranyl acetone) are produced either by decarboxylation or through the microbial oxidation of fatty acids during MLF (Chen et al., 2019; Yu et al., 2022). Ketones, characterized by intense aromas even in low concentrations, could contribute synergistically to the aromatic profile of fermented apple juice (Guiné et al., 2021). Furthermore, acetoin is associated with off‐flavors such as creamy and buttery notes in fermented beverages and ciders (El Hadi et al., 2013; Jagtap & Bapat, 2015; Liu et al., 2020; Qin et al., 2018). Although this aroma compound has a high sensory threshold (150 mg/L; Table 1), it is still an unwanted character in any cider and should be monitored for quality purposes.

Overall, the composition of cider's volatile compounds is attributable to those already present in the apples (e.g., volatile and fatty acids) and those formed during fermentation (e.g., esters and higher alcohols). For cider producers, especially those exploring RFAs, the novelty and unique profile of these apples offer an exciting opportunity to develop distinctive ciders. The inherent composition of RFAs, particularly their phenolic and volatile profiles, provides a different sensory foundation compared to traditional dessert and cider apples. Given the novelty of RFAs in cider production, research focused on understanding the interplay between apple variety, yeast metabolism, and resulting volatile compounds is crucial. Developing predictive models for volatile compound production could greatly assist in selecting optimal fermentation conditions and identifying the most suitable RFA varieties for cider production. This strategic approach would not only support the consistent production of ciders with distinctive sensory qualities but also encourage the exploration of different RFA varieties, expanding the diversity and appeal of ciders in the market.

#### 4.7.3 Yeast strains

Numerous studies have shown that the yeast strain used in cider fermentation influences the aromatic complexity and evolution of volatile compounds, affecting primary (determined by the initial product composition), secondary (created during fermentation), and tertiary aromas (created during product maturation and storage) (Hou et al., 2021; Liu et al., 2020; Lorenzini et al., 2019; Padilla et al., 2016; Rosend, Kuldjärv, Rosenvald, et al., 2019). Multiple yeast strains were reported on, including *S. cerevisiae*, *S. bayanus*, *Hanseniaspora vineae*, *Pachysolen tannophilus*, *M. pulcherrima*, *Hanseniaspora uvarum*, *T. delbrueckii*, *Zygosaccharomyces bailii*, *Schizosaccharomyces pombe*, *L. thermotolerans*, *Issatchenkia orientalis*, *Saccharomycodes ludwigii*, *Hanseniaspora osmophila*, and *Starmerella bacillaris*. The characteristics of the different strains and the interactions between the strains, as well as the combined effects between substances, give cider its complex flavor characteristics (Wu et al., 2023).

However, research has shown that using different yeast strains (*S. bayanus* vs. *S. cerevisiae*) and storage times (0–3 months) decreases the total phenolic content (e.g., through enzymatic reactions and oxidation reactions) in ciders made from both red‐skinned and non‐red‐skinned (e.g., yellow, green, or blush skins) apples (Lachowicz et al., 2019). Moreover, the selected yeast can lead either to the protection of phenolic compounds or to their destruction (Lachowicz et al., 2019). On the contrary, several studies found that yeast strains (*S. cerevisiae* and *S. pombe*) had no effect (*p* > .05) on the total phenolic content in cider and that instead it was strongly influenced by apple variety (He et al., 2021; Riekstina‐Dolge et al., 2014). However, yeast strain selection for modern cider making often fails to account for the potential for high phenolic concentrations in new cider apple varieties (such as RFAs; Section 2.3) that could have inhibitory effects on fermentation rate or influence volatile evolution during fermentation (Cairns et al., 2022).

Ciders possess a vast array of flavor characteristics driven by the yeast strain's ability to produce volatile chemicals via several biosynthetic pathways (Swiegers et al., 2005; Way et al., 2022) (Sections 4.7.1 and 4.7.2). With the advancement of scientific understanding, it will be feasible to isolate particular yeasts and bacteria to generate ciders of any desired style and satisfy evolving consumer preferences (Swiegers et al., 2005). These technologies would especially be useful in the production of ciders from RFAs.

### 4.8 Pasteurization

Pasteurization (Figure 2) is a thermal processing technique that involves heating the cider to eliminate harmful microorganisms and prevent fermentation of residual sugar once it is bottled (Le Quéré et al., 2006; Moulton & Zimmerman, 2010). Not only does the combination of live cultures (i.e., residual active yeast) and fermentable sugar in bottles pose a safety risk by potentially causing bottle explosions but it also leads to the formation of haze, sediment, and unwanted aromas leading to poor cider quality. Pasteurization can be performed as a batch process just before bottling (Heikefelt, 2011) or after bottling (Aguilar‐Rosas et al., 2007; Merwin et al., 2008). Different pasteurization time and temperature combinations are used depending on the product matrix and available equipment. The high‐temperature short‐time (HTST) pasteurization method (76.6–87.7°C for 25–30 s) is commonly used for the thermal treatment of apple juice and generally preferred over low‐temperature long‐time (LTLT) pasteurization (Aguilar‐Rosas et al., 2007; Beech, 1972). The brief exposure to high temperatures minimizes the degradation of heat‐sensitive compounds, maintaining the juice's flavor, color, and bioactive components (Mieszczakowska‐Frąc et al., 2021). Additionally, HTST systems are more energy efficient and allow for higher production rates compared to traditional batch pasteurization methods (Jay et al., 2005).

Although pasteurization is recommended to ensure the safety of a cider (Techakanon & Sirimuangmoon, 2020), the application of any heat can potentially reduce the sensory properties, specifically impacting the color, flavor, and even nutritional quality (i.e., essential nutrients, including sugars, organic acids, vitamins, minerals, and bioactive compounds such as phenolics, which contribute to the health benefits of a product) of the product (Aguilar‐Rosas et al., 2007; Choi & Nielsen, 2005; Heikefelt, 2011; Minatel et al., 2017; Valappil et al., 2009). Compared to HTST, LTLT pasteurization may result in slightly lower degradation of some volatile compounds due to its lower temperature; however, the extended heat exposure can still cause oxidation and breakdown of key aroma and flavor compounds (Odriozola‐Serrano et al., 2009). Therefore, cider producers are often hesitant to pasteurize due to concerns that the conventional heat treatments used for apple juice, such as hot‐fill processes at high temperatures ranging from 88 to 90°C, may compromise the flavor of their unfiltered product (Splittstoesser et al., 1996). Furthermore, high temperatures lead to considerable losses of important volatile compounds (such as higher alcohols and esters) (Williams, 1974) that impact the flavor of the cider (López et al., 2002) and have been shown to cause *oxidized* or *cooked* flavors (Merwin et al., 2008). While it is essential to reduce the risk of microorganism contamination, it is equally important to strike a balance to preserve the quality properties. Therefore, it is important to carefully monitor temperature, time, and free SO_2_ levels to minimize the adverse effects of pasteurization.

Conflicting research shows both beneficial and detrimental effects of pasteurization on cider phenolic compounds. Studies focusing on the positive effects have shown that the high temperatures during pasteurization inactivate PPO, reducing the oxidation of phenolic compounds and subsequent browning (Choi & Nielsen, 2005; Merwin et al., 2008; Nicolas et al., 1994). Heat treatment causes oxidation and hydrolysis of phenolic glycosidic and ester bonds, releasing aglycones. As apple juice production involves interactions between polysaccharides and phenolic compounds, some bound phenolics are released during thermal treatment (Feng et al., 2021). Inactivation of PPO and this release explain the increase in phenolic compounds with elevated temperatures, such as during pasteurization.

Nonetheless, previous research shows that phenolic compounds oxidize during pasteurization, potentially negatively affecting the sensory qualities of cider (Wolfe & Liu, 2003). Phenolic compounds contribute to the specific odor and flavor qualities in apples and apple products; thus, any losses impact the functional and sensory properties of ciders. Several studies report considerable phenolic losses in thermally treated apple juice (Aguiar et al., 2012; Chen et al., 2013; Choi & Nielsen, 2005). Yuste et al. (2020) found that anthocyanins in RFAs degraded more during pasteurization in a tubular system (10 min at 94°C) than other phenolic compounds. While fermentation reduces browning by lowering oxygen levels and altering pH (inhibiting PPO activity), browning can still occur in ciders even postpasteurization (Février et al., 2017). Sulfites as preservatives help mitigate browning by binding to oxygen and limiting oxidation (Merwin et al., 2008).

Alternative nonthermal methods of pasteurization aim to ensure product safety while preserving the cider's sensory properties as compared to the original liquid (i.e., juice or cider) (Aguilar‐Rosas et al., 2007; Evrendilek et al., 2000; Minatel et al., 2017). Novel technologies such as high‐intensity pulsed electric field, membrane filtration, high‐pressure processing, ultraviolet technology, cold plasma, and ultrasound have been investigated, with some adopted by the beverage industry (Bhat & Sharma, 2016; Călugăr et al., 2021; Chakraborty et al., 2015; Chen et al., 2013; Illera et al., 2019; Minatel et al., 2017; Ozen et al., 2022). While effective in inactivating microorganisms, their impact on the flavor of RFA ciders and other sensory properties is limited (Valappil et al., 2009). Nevertheless, combining these techniques, known as hurdle technology, could mitigate the drawbacks associated with the individual techniques and improve efficacy in eradicating microorganisms while preserving the nutritional integrity of fruit juice/cider (Chen et al., 2013). Further research is required to identify cost‐effective treatments that ensure a high‐quality cider when utilizing RFAs.

## 5 CONCLUSIONS

As the cider industry grows rapidly, targeted research in apple biochemistry, sensory properties, processing, and fermentation is increasingly needed. Although many publications explore the physicochemical and sensory properties of dessert, cider, and RFAs, along with their utilization in juicing and cider production, these works are scattered and lack a systematic overview. Given the complexity of cider production, regulating flavor and aroma is crucial and requires a deeper understanding of how specific apple varieties shape the final product.

RFAs hold strong potential for cider production due to their naturally high phenolic content and distinct sensory properties. However, their elevated anthocyanin concentrations present challenges in stability, degradation, and potential browning during cider processing and storage. Despite increasing interest in RFAs, knowledge gaps remain, particularly regarding the relationship between phenolic composition and sensory perception, organic acids’ role in consumer acceptance, and fermentation's impact on aroma and flavor.

Future research should refine analytical techniques for phenolic compounds (specifically tannins) to establish clearer sensory correlations. Predictive modeling of volatile compound formation during fermentation could enhance flavor consistency, optimize yeast selection, and tailor fermentation strategies for RFAs. Alternative cider‐making techniques, such as MLF, warrant investigation to modulate acidity and enhance sensory complexity. Understanding anthocyanin transformations during juice processing, fermentation, and storage is essential to improving color stability in RFA ciders.

Addressing these gaps will equip cider producers to optimize processing techniques, enhance stability and sensory quality, and boost RFA cider marketability. A structured research approach will maximize RFA potential, driving the creation of high‐quality, distinct ciders aligned with evolving consumer preferences.

## AUTHOR CONTRIBUTIONS

**Marbi Schwartz**: Conceptualization; investigation; project administration; visualization; writing—review and editing; writing—original draft. **Dalene de Beer**: Conceptualization; writing—review and editing; supervision; visualization. **Jeannine Marais**: Conceptualization; project administration; funding acquisition; supervision; visualization; writing—review and editing.

## CONFLICT OF INTEREST STATEMENT

The authors declare no conflicts of interest.
